# Epidemiology of *Schistosoma mansoni* infection in Ituri Province, north-eastern Democratic Republic of the Congo

**DOI:** 10.1371/journal.pntd.0009486

**Published:** 2021-12-02

**Authors:** Maurice M. Nigo, Peter Odermatt, Georgette B. Salieb–Beugelaar, Oleksii Morozov, Manuel Battegay, Patrick R. Hunziker

**Affiliations:** 1 Nanomedicine Translation Group, Intensive Care Unit, University Hospital Basel, University of Basel, Basel, Switzerland; 2 CLINAM–European Foundation for Clinical Nanomedicine, Basel, Switzerland; 3 University of Basel, Basel, Switzerland; 4 Institut Supérieur des Techniques Médicales (ISTM) Nyankunde, Bunia, Democratic Republic of Congo; 5 Swiss Tropical and Public Health Institute, Basel, Switzerland; 6 Department of Infectiology & Hospital Hygiene, University Hospital Basel, Basel, Switzerland; Federal University of Agriculture Abeokuta, NIGERIA

## Abstract

**Background:**

Schistosomiasis, caused by *Schistosoma mansoni*, is of great significance to public health in sub–Saharan Africa. In the Democratic Republic of Congo (DRC), information on the burden of *S*. *mansoni* infection is scarce, which hinders the implementation of adequate control measures. We assessed the geographical distribution of *S*. *mansoni* infection across Ituri province in north-eastern DRC and determined the prevailing risk factors.

**Methods/Principal findings:**

Two province–wide, community–based studies were conducted. In 2016, a geographical distribution study was carried out in 46 randomly selected villages across Ituri. In 2017, an in–depth study was conducted in 12 purposively–selected villages, across the province. Households were randomly selected, and members were enrolled. In 2016, one stool sample was collected per participant, while in 2017, several samples were collected per participant. *S*. *mansoni* eggs were detected using the Kato–Katz technique. In 2017, a point–of–care circulating cathodic *S*. *mansoni* antigen (POC–CCA) urine test was the second used diagnostic approach. Household and individual questionnaires were used to collect data on demographic, socioeconomic, environmental, behavioural and knowledge risk factors.

Of the 2,131 participants in 2016, 40.0% were positive of *S*. *mansoni* infection. Infection prevalence in the villages ranged from 0 to 90.2%. Of the 707 participants in 2017, 73.1% were tested positive for *S*. *mansoni*. Prevalence ranged from 52.8 to 95.0% across the health districts visited. Infection prevalence increased from north to south and from west to east. Exposure to the waters of Lake Albert and the villages’ altitude above sea level were associated with the distribution.

Infection prevalence and intensity peaked in the age groups between 10 and 29 years. Preschool children were highly infected (62.3%). Key risk factors were poor housing structure (odds ratio [OR] 2.1, 95% 95% confidence interval [CI] 1.02–4.35), close proximity to water bodies (OR 1.72, 95% CI 1.1–2.49), long-term residence in a community (OR 1.41, 95% CI 1.11–1.79), lack of latrine in the household (OR 2.00, 95% CI 1.11–3.60), and swimming (OR 2.53, 95% CI 1.20–5.32) and washing (OR 1.75, 95% CI 1.10–2.78) in local water bodies.

**Conclusions/Significance:**

Our results show that *S*. *mansoni* is highly endemic and a major health concern in Ituri province, DRC. Infection prevalence and intensity, and the prevailing socioeconomic, environmental, and behavioural risk factors in Ituri reflect intense exposure and alarming transmission rates. A robust plan of action is urgently needed in the province.

## Author summary

Intestinal schistosomiasis threatens many people in the tropical world, particularly those in Sub–Saharan Africa. Information on schistosomiasis in the Democratic Republic of Congo (DRC) is very scarce, which is a major barrier to planning and implementing efficient control programmes. We conducted two community–based studies with the objective of assessing the geographical distribution of *S*. *mansoni* infection across the Ituri province in north-eastern DRC and determining the prevailing risk factors. In 2016, a geographical distribution and in 2017, an in–depth studies were conducted in selected villages. Households were randomly selected and members, aged one year and older and present on the survey day, were enrolled. In 2016, one stool sample was examined per participant, whereas several stool samples were examined for each participant in 2017. *S*. *mansoni* eggs were detected using the Kato–Katz technique. A point–of–care circulating cathodic *S*. *mansoni* antigen (POC–CCA) urine test was also used in 2017. Household and individual questionnaires were used to collect demographic, socioeconomic, environmental, behavioural and knowledge data. In 2016, of the 2,131 participants, 40.0% of were infected with *S*. *mansoni*. Of the 707 participants in 2017, 73.1% were tested positive for *S*. *mansoni*. Infection prevalence increased from north to south and from west to east. Exposure to the Lake Albert and villages’ altitude were main drivers of the distribution. Men and women had the same infection risk. Infection prevalence and intensity peaked in the age groups between 10 and 29 years. Preschool children were highly infected. Poor housing structure, proximity to water bodies, long–term residence in a community, lack of latrine in the household, and swimming and washing in local water bodies were the main risk factors. Our results confirm that *S*. *mansoni* is highly endemic in Ituri province, DRC. Both infection prevalence and intensity indicate intense exposure and alarming transmission. Control interventions are warranted and should pay attention to high–risk communities and population groups, including preschool children.

## Introduction

Schistosomiasis is a major cause of global disability, morbidity, and mortality [1, 2]. Recent estimates suggest that nearly 800 million people are at risk for schistosomiasis, while 240 million people are infected [3]—more than 90% of whom live in sub–Saharan Africa (SSA) [4]. Population-based preventive chemotherapy (PCT) using praziquantel (PZQ) is the recommended control strategy [5].

Schistosomiasis is a water-borne parasitic disease caused by trematode species of the genus *Schistosoma*. In SSA, there are four predominant species, namely *S*. *mansoni*, which causes intestinal schistosomiasis; *S*. *haematobium*, the agent of genitourinary schistosomiasis; *S*. *intercalatum;* and *S*. *guineensis* [6]. *Schistosoma* species exhibit an indirect life cycle with one intermediate host. Parasite eggs are released into the environment (i.e., local water bodies) via the stools or urine of infected people. Transmission of the parasite during its infective stages is multifactorial and depends on the environment, parasite, vector, and host [7]. Human water contact patterns critically influence transmission and are associated with the socio-economic and behavioural characteristics of a local population, such as lack of proper sanitation, limited access to safe water, and poor hygiene (WaSH).

The Democratic Republic of Congo (DRC) has the third highest number of reported schistosomiasis cases in SSA (15 million) after Nigeria (29 million) and the United Republic of Tanzania (19 million) [8]. Three species of *Schistosoma* have been reported in DRC: *S*. *mansoni*, *S*. *haematobium*, and *S*. *intercalatum*. *S*. *mansoni* is abundant in the eastern region of the country, along the shores of the great lakes, and in the western region. *S*. *haematobium* is present in the central and south-eastern regions, while *S*. *intercalatum* can be found in the central-northern regions of the country [9].

In the DRC, knowledge on the current burden of this neglected tropical disease (NTD) including schistosomiasis is lacking [10]. The DRC suffers from a recent history of war and ongoing tribal and armed conflicts, the results of which have led to economic deterioration, severe poverty, and badly functioning health services. Data from the available large surveys are more than twenty–years old [9, 11]. Existing publications reporting on *Schistosoma* infection focus on Belgian military personnel returning from DRC to Belgium [12] or describe epidemiological studies carried out in a few areas of the country [9], such as Kinshasa [13–16], the capital city, and in the provinces of Kongo Central [17–19], Bandundu [20] Kwilu [21, 22], Kasaï Central, Kasaï Oriental [11], Maniema [23–28], South–Kivu [29], Katanga [30, 31] and Haut-Uele [32].

Publications relating to the north-eastern provinces are scarce [24, 29, 32] or date back to the colonial period [9, 28]. In 2012, the Ministry of Health (MOH) launched a national survey of NTDs and a new strategic plan was elaborated in 2016 [33, 34]. Although a national plan against NTDs, including schistosomiasis, has been implemented by the Ministry of Health, the intervention strategy is based on outdated prevalence data in many regions. For example, the strategy in place in Ituri province in north-eastern DRC is applicable to moderate–risk settings (10–49%), yet Ituri appears to be a high–risk area [32]. In this situation, numerous infected and untreated people may sustain transmission cycles, so long as treatment coverage remains inadequate [35]. Thus, there is an urgent need to provide updated knowledge to national and provincial health authorities about the distribution of intestinal schistosomiasis as a first step towards developing and implementing appropriate interventions. This study therefore assessed the prevalence, intensity, and associated risk factors for *S*. *mansoni* infection in the province, north-eastern DRC.

## Materials and methods

### Ethics statement

This study was approved by the Swiss Ethical Commission (Ethikkommission Nordwest und Zentralschweiz [EKNZ], Ref. No. UBE–15/78) and the University of Kisangani’s Research Ethical Commission (Ref No: CER/003/GEAK/2016). Research authorization was granted by the Nyankunde Higher Institute of Medical Techniques (Ref No 70/ISTM–N/SGAC/2017), Bunia, DRC. Permission for field work was obtained from Ituri Provincial Health Division (Ref. 054/433/DPS/IT/06/2016 and Ref. 054/472/DPS/IT/06/2017) and from all relevant health districts. Prior to enrolling study participants, the study objectives and procedures were explained in the local language to each prospective participant. Written informed consent was obtained from all study participants aged 15 years or older. Adolescents (15–17 years) signed the consent forms in the presence of their parents/guardians and the village health volunteer. Parents or legal guardians signed assent forms for participants under the age of 15. At the end of the study, all participants received Mebendazole (500 mg, single dose) for general deworming, and those diagnosed with *S*. *mansoni* infection were treated with praziquantel (40mg/kg) in accordance with the DRC national deworming guidelines [36].

### Study area

**Fig 1:**
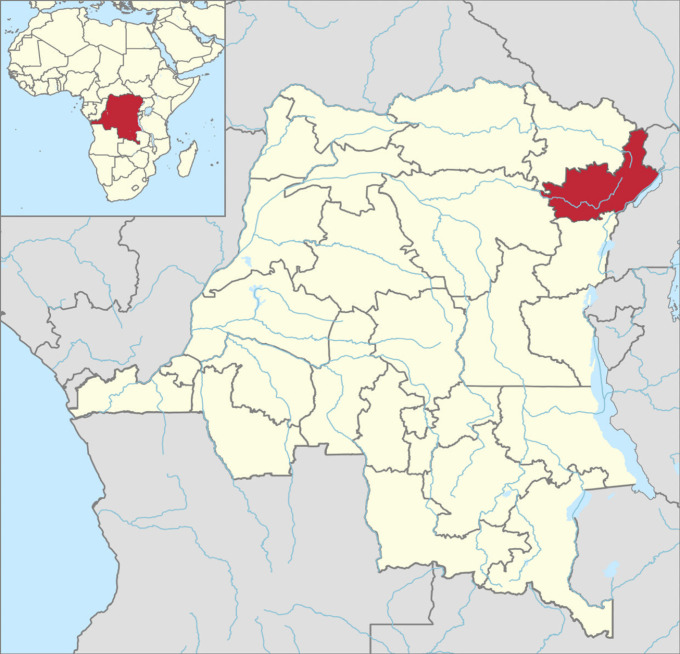
Study site: Ituri province map in the north-eastern of DRC. Map of the north-eastern of the Democratic Republic of the Congo in the centre of Africa, and the Ituri province. Map created by Oleksii Morozov based on geo data obtained from https://data.humdata.org/dataset/f42132b9-8cc6-4201-b020-9259c56e8868/resource/e335bc0b-b946-437f-b771-beb5599eaf1d/download/cod_admbnda_rgc_itos_20190911_shp.zip and https://www.naturalearthdata.com.

Ituri province is situated in north-eastern DRC and has a surface area of 65,658 km^2^ (Fig 1). It is divided into counties, territories (Aru, Mahagi, Djugu, Irumu, and Mambasa), and 36 health districts. The province is heavily irrigated by natural water streams and is characterized by great geographical and demographic diversity. Aru territory, in the north, is a plateau about 1,100 m above sea level and covered by a combination of wooded areas, grassy savannah, and forest galleries. Mahagi territory has a vegetation profile similar to that of Aru, and a peak altitude of around 1,800 m, followed by a steep slope that descends to Lake Albert in the east. Djugu territory boasts high hills (up to 2,300 m above sea level in the Blue Mountains) and is mainly covered by grassy savannah, with a few forest galleries in the west. The mountains slope steeply towards Lake Albert in the east. Irumu territory has geographic characteristics similar to Djugu in the east and is covered with dense forest in the west. Mambasa territory is a lowland region, completely covered with dense equatorial forest.

Health districts throughout the territories are delineated based on population size. All of the health districts offer curative and preventive services such as vaccination, infant and prenatal clinics, deliveries, and human immunodeficiency virus (HIV) testing. Some health districts engage in tuberculosis and leprosy care, and in community control activities. Laboratory diagnosis for diseases like malaria and soil-transmitted helminths are available in most of the general district hospitals, but many health centres lack diagnostic tools, personnel and/or consumables. Praziquantel is available in all of the district hospitals bordering Lake Albert. However, drugs for most neglected tropical diseases (NTDs) are only sporadically provided to some health districts by the provincial branch of the national control programme.

About 5.3 to 9.0 million people of Sudanese, Nilotic, Bantu, Nilo–Hamite, and Pygmy ethnicities live in Ituri province [37]. Approximately 91% of the population lives in the northern and eastern areas of the province. Both rural and urban populations engage in subsistence farming, including livestock rearing. Cattle breeding, for example, is particularly practiced in Djugu, Irumu, Mahagi and Aru territories. Young people are involved in the artisanal exploitation of minerals such as gold, diamonds and coltan. Fishing activities are important to lakeside communities, while populations close to forested areas (Mambasa, in particular) harvest timber for commercial and domestic purposes. These occupations, coupled with the general lack of safe water, frequently expose Ituri’s population to *S*. *mansoni* contaminated streams and lakes.

For more than two decades, Ituri province has been subject to war, turmoil, and social conflict [38]. The socioeconomic situation in Ituri province is challenging, with a high degree of poverty. The DRC ranked 176^th^ out of 188 countries included in the Human Development Index in 2017 [39]. In 2011, a Water and Sanitation Program (WSP) strategic overview estimated that 50 million Congolese (75.0%) did not have access to safe water, while approximately 80–90% did not have access to improved sanitation [40]. Likewise, the UNICEF/WHO 2017 database showed that in 2015, 84.0% of DRC’s rural population had no hygiene facility, 45.3% had unimproved sanitation, 10.2% resorted to open defecation, 53.0% used unimproved water sources, and 16.0% used surface water [41]. According to data from the Food and Agricultural Organization (FAO), Ituri province measures 0.4 to 0.45 on the normalized difference vegetation index (NDVI) [42].

### Study design and population

**Fig 2:**
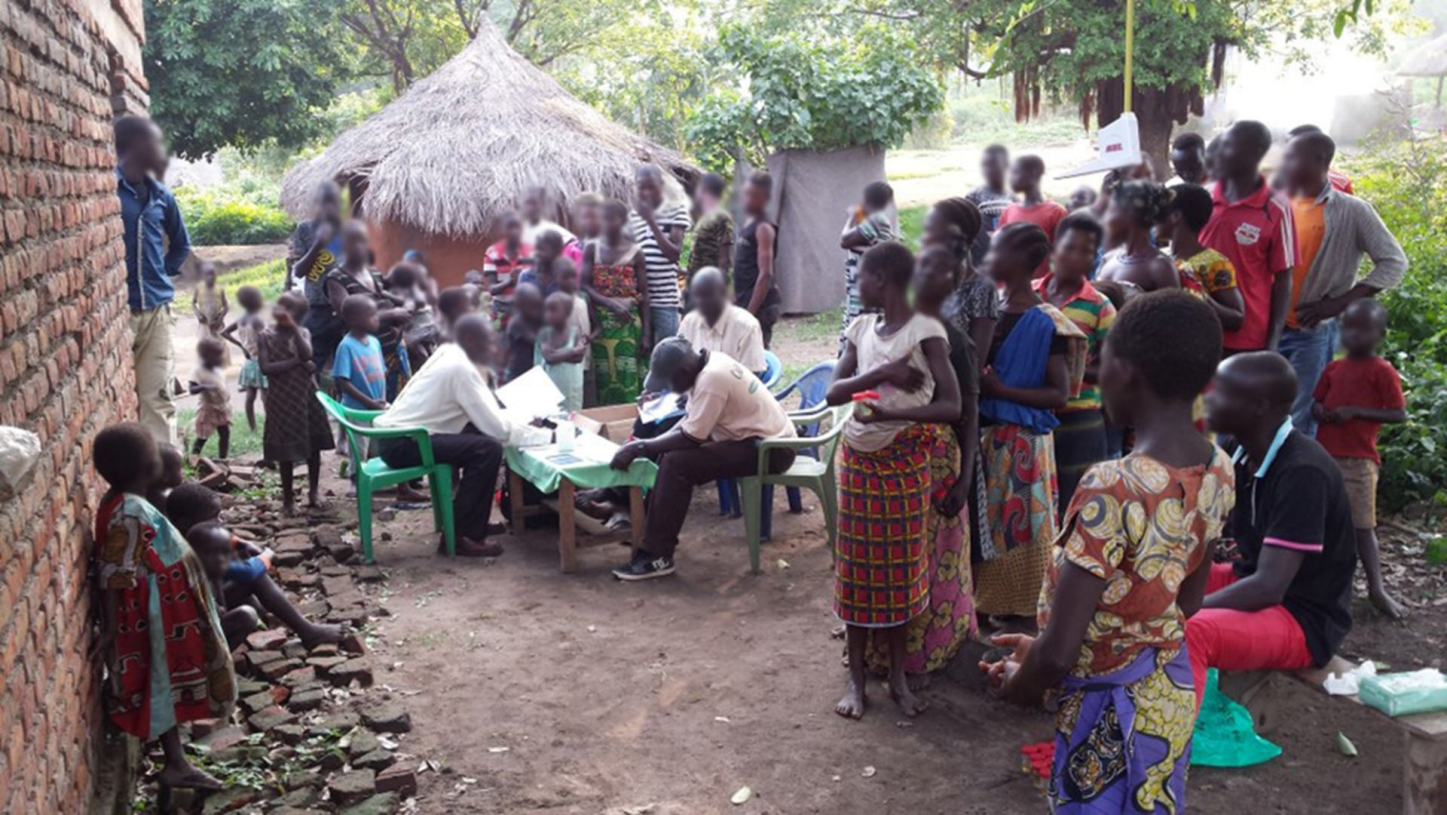
Study field procedures. Enrolment of study participants, administration of household and individual questionnaires, anthropometric measurements, and collection of stool samples.

In 2016 (June–August) and 2017 (July–September), during the short dry season, two community–based studies were carried out in selected villages in Ituri province (Fig 2). In 2016, a cross–sectional study was carried out in 46 randomly-selected villages to assess the geographical distribution of *S*. *mansoni* infection prevalence and infection intensity. In 2017, an in–depth, cross–sectional study was conducted in 12 purposively-selected villages to identify the risk factors for infection. One village (Pekele) was included in both years. For both studies, people aged one year and older were eligible to participate.

The 2016 cross–sectional study used a multi-stage random sampling procedure. Twelve health districts were randomly selected across the province according to eco-epidemiological criteria (urban/rural, proximity to the lake, altitude, vegetation). Several villages were randomly selected in densely populated districts, while fewer villages were randomly selected in sparsely populated districts.

In both studies, unique reference codes were assigned to each study participant.

### Assessment of *S*. *mansoni* infection

In 2016 and in 2017, participants received plastic specimen cups with lids to collect stool samples. The cups had been labelled with names and codes the day before collection. Participants and/or parents were asked to collect the first morning sample. The laboratory technicians collected the stool samples between 6:00 a.m. and 8:00 a.m. and took them to the health facility laboratory. Some participants who did not have a sample ready at the agreed-upon time were asked to take their samples to the health facility laboratory themselves.

In 2016, study participants provided one stool sample from which *S*. *mansoni* could be diagnosed using Kato–Katz test [43]. About one hour after collection, stool specimens were transported and examined in the laboratory of the health facility serving the village. Two Kato–Katz smears were prepared for each stool sample [43]. The smears were allowed to clear for approximately half an hour before being read. The number of eggs detected on each smear was recorded for each helminth species separately.

In 2017, participants provided one stool sample over five consecutive survey days. On the last day, participants were asked to provide a urine sample as well for a point–of–care cathodic circulating antigen (POC–CCA) test to detect *S*. *mansoni*. Both stool and urine samples were transported to the health facility laboratory for processing within an hour of collection. A duplicate Kato–Katz test [43] was performed (2 smears per stool) on each stool sample. Again, the number of eggs detected on the smear was recorded for each helminth species separately. In addition, a *S*. *mansoni* POC–CCA test [44] was carried out on the urine samples.

To ensure quality in both surveys, about 20% of the Kato-Katz slides were re-read by the principal investigator. In practice, every tenth negative and every tenth positive slide were put aside for re-examination. There were some small differences in egg counts when the number of eggs was high. In these cases, the microscopist repeated his count and, if the same as before, we took the mean value of the microscopist’s two counts and that of the principal investigator. Trace and weakly positive results from the urine POC-CCA test were discussed among the laboratory team and the principal investigator.

### Questionnaire data

In 2016, demographic information was collected using a short questionnaire addressed to each study participant. However, in 2017, two questionnaires—a household and an individual questionnaire—were administered to the head of household. The household questionnaire included questions regarding the number of household members, the availability and quality of sanitation (latrine), the source and type of water used, the building material of the house, goods of the house, estimated monthly income, and the existence of mass drug administration (MDA) for the benefit of the villagers. The individual questionnaire addressed demographic details such as age, sex, tribal group, religion, main occupation, education, usual defecation place, knowledge of schistosomiasis, its transmission patterns, and prevention and treatment options. Information about potential risk factors, such as exposure to water, socioeconomic status, time of residence, lack of sanitation and safe drinking water, was also collected.

### Data management

In both studies, data were entered in Excel and cross checked with the source data. Data management and data analysis were performed with Stata, version 14.2 (Stata Corp LP; College Station, USA). Age groups were defined as follows: (i) 1–4 years, (ii) 5–9 years, (iii) 10–14 years, (iv) 15–19 years, (v) 20–29 years, (vi) 30–39 years, (vii) 40–49 years, and (viii) ≥50 years. Age categories were chosen in such a way as to differentiate the level of infection among infants, children, younger and older adolescents, young adults, adults, and older people. Body mass index (BMI) was calculated, and four BMI categories were established: underweight (<18.5 kg/m^2^), normal weight (18.5–24.9 kg/m^2^), overweight (25.0–29.9 kg/m^2^), and obese (≥30 kg/m^2^). Using the arithmetic mean of the egg positive stool samples, *S*. *mansoni* infection intensity (eggs per gram [epg]) was categorized as light (1–99 epg), moderate (100–399 epg), and heavy (≥400 epg) [43, 45].

### Statistical analysis

Descriptive statistics (means and frequencies) were used to summarize continuous and categorical variables, respectively. The chi–square test (χ^2^) and Fisher exact test were used to compare proportions. A univariate logistic regression analysis was performed to associate *S*. *mansoni* infection (outcome) with potential risk factors (predictors), such as demographic, geographical, behavioural, and socioeconomic variables. Co–variables exhibiting an association at a significance level of at least 20%, as determined by the likelihood ratio test (LRT), were included in the multivariate logistic regression models. Odds ratios (OR) and adjusted OR (aOR) for multivariate analysis and 95% confidence intervals (95% CI) were calculated. To visualize prevalence rates at health district and village levels, a two-way scatter bar command, which displays numeric (y, x) data as histogram–like bars, was performed using the vertical bar plot option in STATA. Bars were drawn at the specified xvar values (health districts and villages) and extended up from 0 according to the corresponding yvar values (prevalence). To explore the relationship between *S*. *mansoni* infection risk and age, age–and sex–prevalence curves were produced using the twoway quadratic fitted values. This command calculates the prediction for yvar (prevalence or intensity) from a linear regression of yvar on xvar and xvar^2 and plots the resulting curve. An *S*. *mansoni* distribution map was made as follows: village prevalence (resulting from the Kato–Katz tests in 2016 and 2017) and geographic decimal coordinates (latitude and longitude) were entered in two adjoining columns in Excel and then plotted. A non–uniform spline interpolation was performed with MATLAB, revealing shadows. The intensity of the colour was proportional to prevalence levels. Dark coloured shadows indicate high prevalence and lighter shadows (high transparency) indicate low prevalence. All *p–*values below 5% were considered statistically significant.

## Results

### Study population

**Fig 3:**
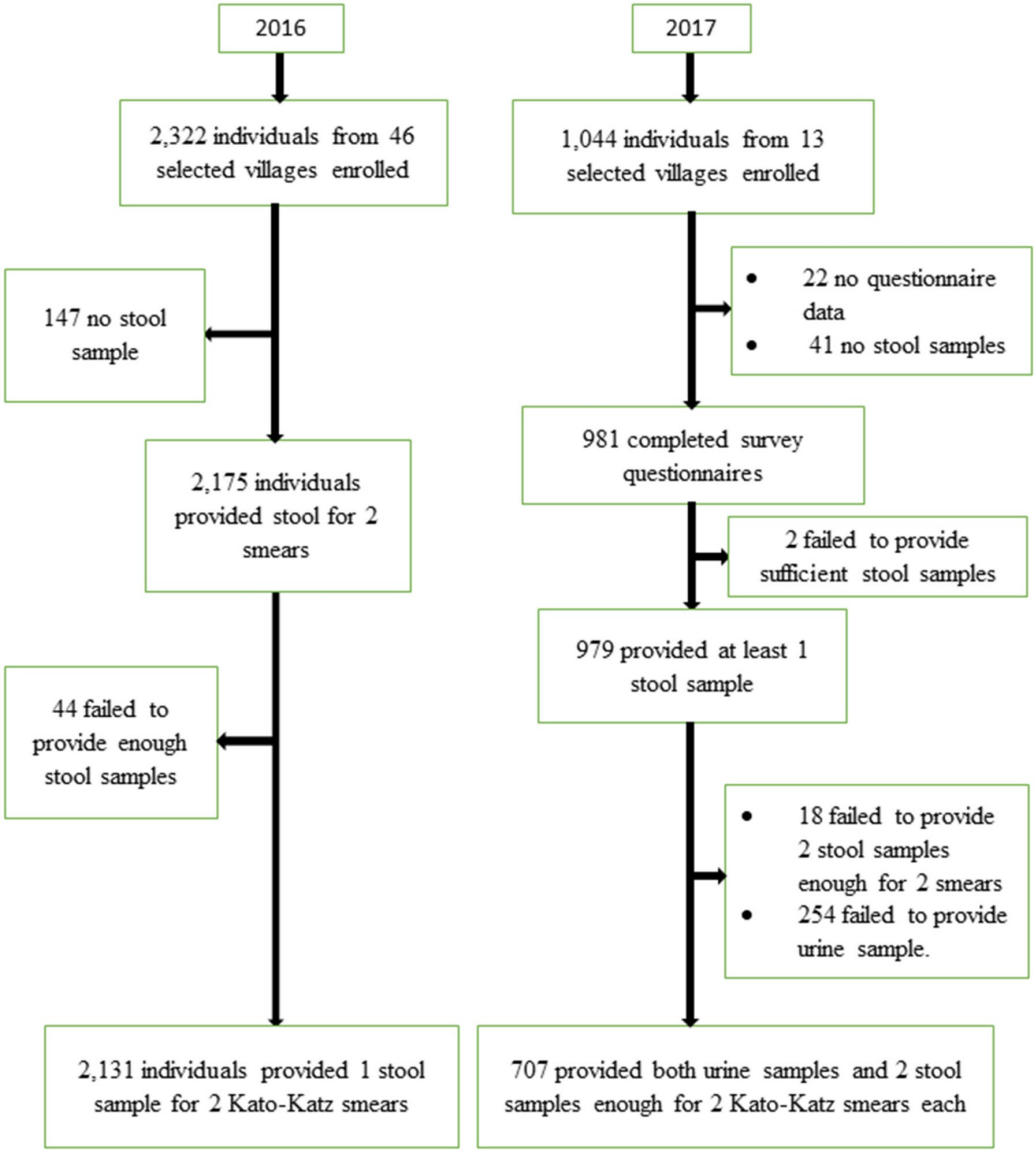
Study participants’ inclusion flowchart for the Ituri schistosomiasis surveys. Left: In 2016, geographical distribution study in 46 villages; Right: In 2017, in–depth study in 12 villages.

A total of 3,366 participants were enrolled in the study [2,322 in 2016 and 1,044 in 2017]. In 2016, 2,131 (91.8%) participants completed all examinations and were included in the final analysis. Similarly, in 2017, a total of 707 (67.7%) of 1,044 enrolled individuals completed all procedures and were included in the final analysis (Fig 3).

Table 1 shows the demographic characteristics of participants in Ituri province. The mean age was 21.6 and 21.0 years in 2016 and 2017, respectively. Female participants outnumbered males both in 2016 (51.0%) and in 2017 (56.3%). Almost half of the participants (46.6%) were younger than 15 years. Participants came from 58 villages in Ituri province. All five ethnic groups, from both rural and urban areas, were represented in the study.

**Table 1:** Demographic characteristics of the participants in Ituri province in 2016 and 2017.

Characteristics		Year of the study		
		Total n (%)	2016 n (%)	2017 n (%)
Overall n (%)		2,838 (100)	2,131 (75.1)	707 (24.9)
No of villages		58 (100)	46 (79.3)	12 (20.7)
No of households		NA	NA	144
Sex	Female	1,485 (52.3)	1,087 (51.0)	398 (56.3)
	Male	1,353 (47.7)	1,044 (49.0)	309 (43.7)
Mean Age (years)		21.6	22.2	21.0
Age categories (years)	1–4	87 (3.1)	26 (1.2)	61 (8.6)
	5–9	594 (20.9)	436 (20.5)	158 (22.4)
	10–14	640 (22.6)	496 (23.3)	144 (20.4)
	15–19	309 (10.9)	241 (11.3)	68 (9.6)
	20–29	340 (12.0)	337 (15.8)	83 (11.7)
	30–39	311 (11.0)	236 (11.1)	75 (10.6)
	40–49	255 (9.0)	199 (9.3)	56 (7.9)
	≥50	222 (7.8)	160 (7.5)	62 (8.8)
Residence	Rural	2328 (82.0)	1 951 (91.6)	377 (53.3)
	Urban	510 (18.0)	180 (8.4)	330 (46.7)
Ethnic groups	Nilo–Hamites	266 (9.4)	106 (5.0)	160 (22.6)
	Bantu	710 (25.0)	398 (18.7)	312 (44.1)
	Nilotic	919 (32.4)	753 (35.3)	166 (23.5)
	Sudanese	651 (22.9)	582 (27.3)	69 (9.8)
	Pygmies	292 (10.3)	292 (13.7)	0 (0)

Note: No (number); NA (not applicable).

### *Schistosoma mansoni* infection prevalence and intensity

Table A in S1 Text summarizes the infection prevalence of the two surveys according to the different diagnostic approaches. In 2016, using 1KK, the prevalence was 40.0% (95% CI 37.9–42.1); in 2017, it was 38.5% (95% CI 34.9–42.1). Using 1KK, infection intensity was 207.4 epg in 2016 and 100.9 epg in 2017.

Detailed analyses of the results by health district, village, sex, age group, residence, ethnic group, and altitude are shown in Tables B-M in S1 Text.

### Prevalence

In 2016, prevalence ranged from 3.9% to 80.2% across the 12 health districts (*p*<0.001). Infected individuals were found in 43 of the 46 (93.5%) villages investigated. Males were more frequently infected than females (*p* = 0.015). The differences in infection prevalence across the eight age groups were highly significant (*p*<0.001).

The differences in infection prevalence according to altitude, residence, and ethnic group were highly significant for both sexes (*p*<0.001).

In 2017, infection prevalence varied significantly across health districts and villages (*p*<0.001). Infected individuals were found in all 12 (100%) villages investigated. Males had slightly higher infection rates than females (*p* = 0.603). The differences in infection prevalence across the eight age groups were highly significant (*p*<0.001) and were observed among both females (*p* = 0.030) and males (*p* = 0.001).

**Fig 4:**
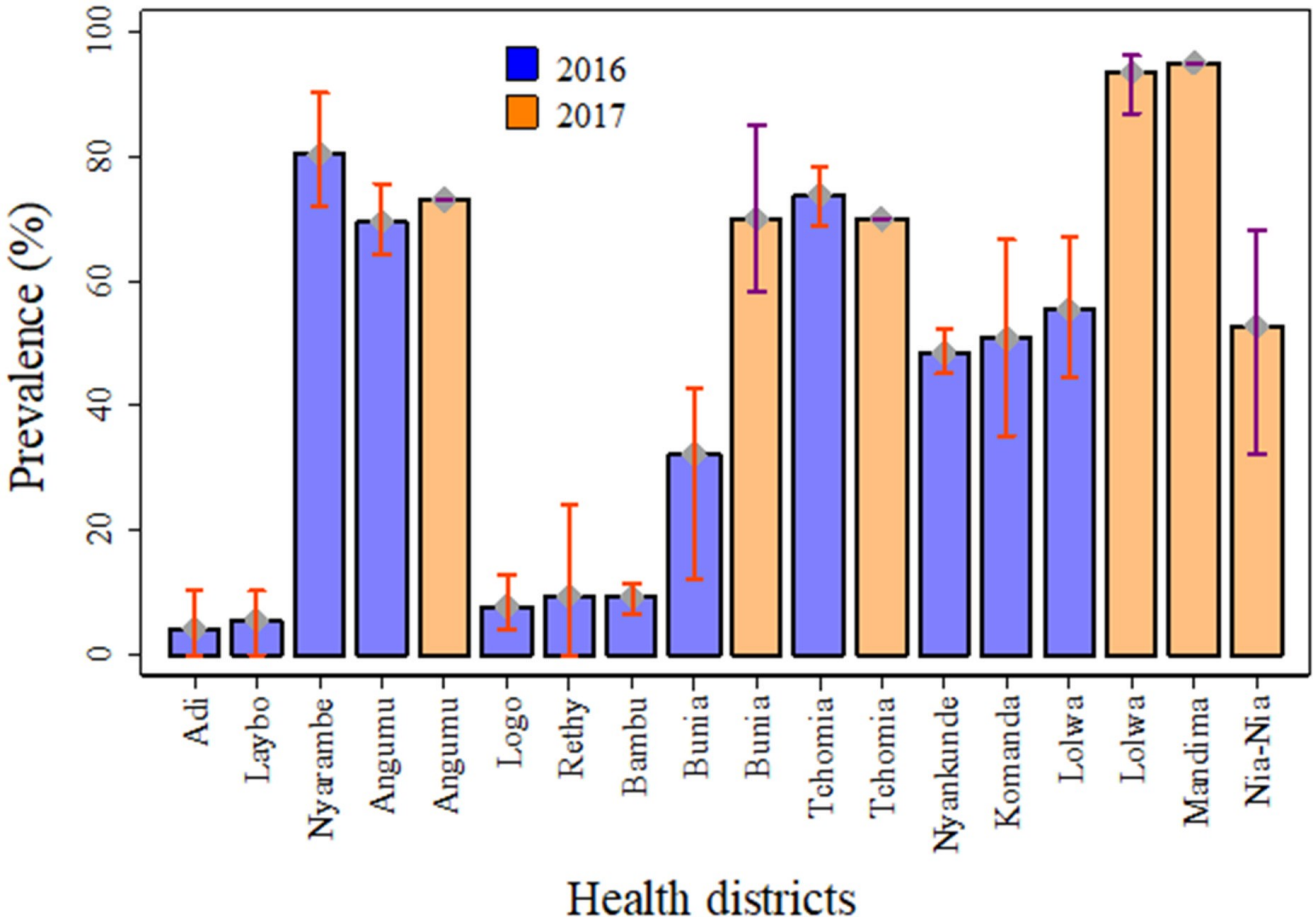
*S*. *mansoni* prevalence by health district in 2016 and 2017. Blue bars: 2016 study (46 study villages across 12 health districts), Kato–Katz test (two smears) of one stool sample. Orange bars: 2017 study (12 study villages across 6 health districts), Kato–Katz test of two stool samples (four smears) and one POC–CCA urine test.

**Fig 5:**
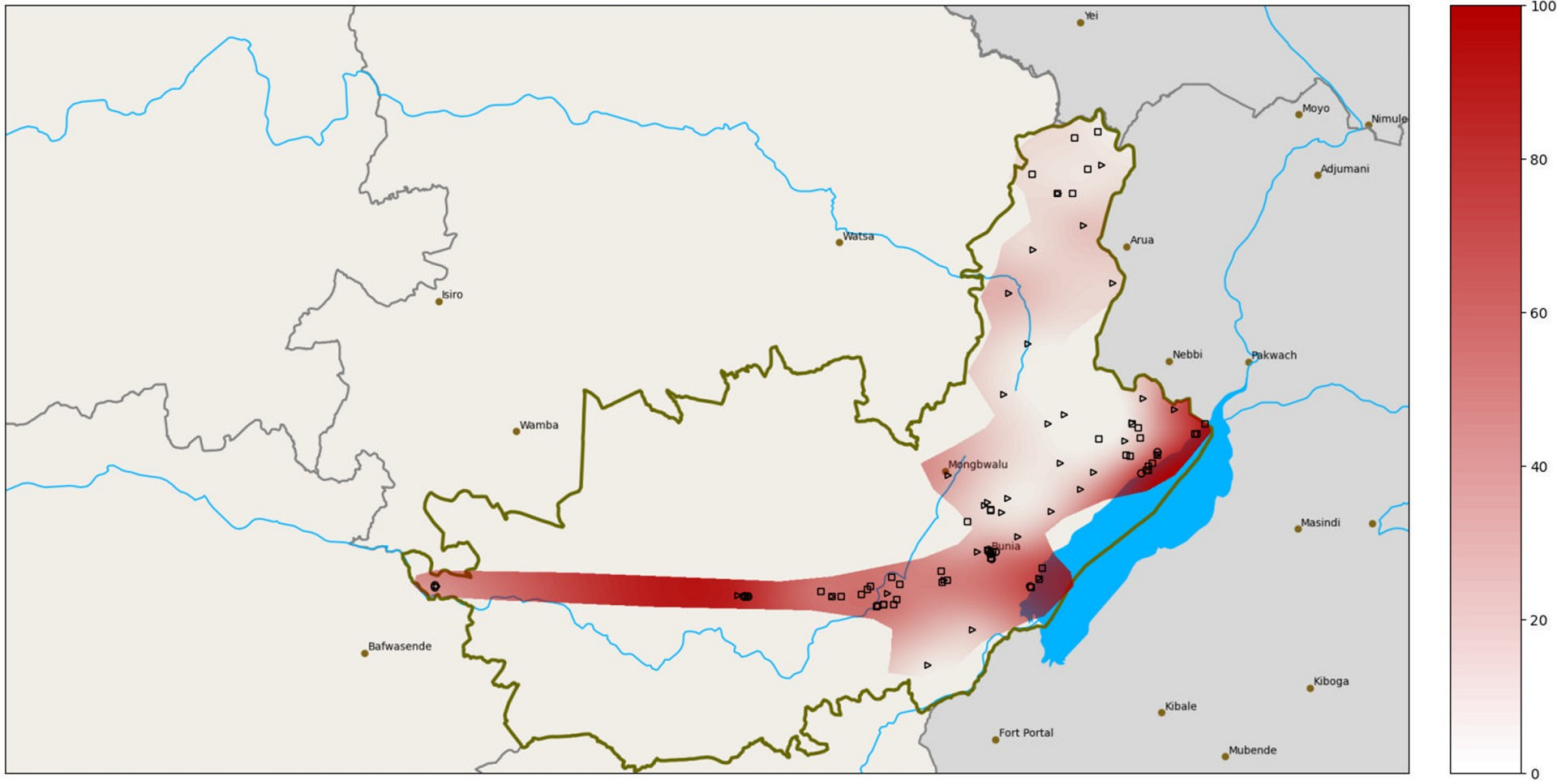
Map of Ituri province with estimated *S*. *mansoni* prevalence. Predicted *S*. *mansoni* prevalence using non–uniform spline interpolation. Intensity of red shadows is proportional to prevalence levels (y-axis). Dots indicate studied villages. Areas outside of the red shadow were inaccessible, dense tropical forest and sparsely populated. Map created by Oleksii Morozov based on geo data from https://data.humdata.org/dataset/f42132b9-8cc6-4201-b020-9259c56e8868/resource/e335bc0b-b946-437f-b771-beb5599eaf1d/download/cod_admbnda_rgc_itos_20190911_shp.zip and https://www.naturalearthdata.com.

Comparing the prevalence of *S*. *mansoni* infection at the health district level (Fig 4), there were considerable higher prevalences in Bunia and Lolwa between 2016 and 2017. Fig 5 shows the *S*. *mansoni* village prevalence distribution in Ituri province in the 2016 and 2017 surveys. The infection prevalence were generally higher in the South than in the North and were highest in the lowlands and along the shores of Lake Albert.

### Infection intensity

The arithmetic mean intensity of *S*. *mansoni* infection in the 12 health districts investigated in 2016 and in the six investigated in 2017 are shown in Tables D and E in S1 Text, respectively. The infection intensities ranged from 0 to 14,424 epg in 2016 and from 60 to 5,472 epg in 2017.

**Fig 6:**
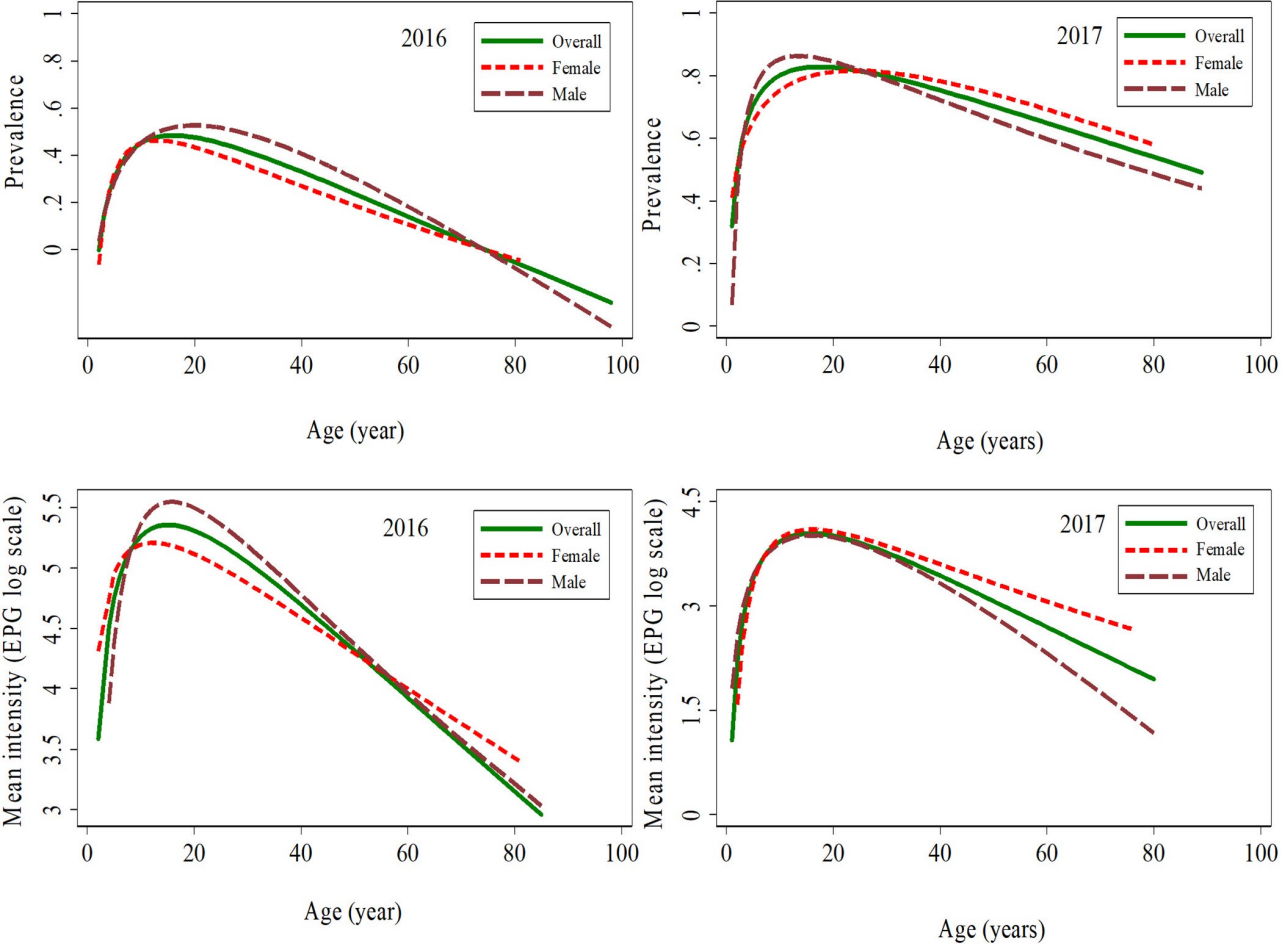
*Schistosoma mansoni* infection prevalence and intensity by age and sex in the two studies. Top: *S*. *mansoni* infection prevalence by age in 2016 and 2017. Bottom: *S*. *mansoni* infection intensity (log scale) by age in 2016 and 2017. Lines: Green (solid): all participants; Red (dashed): female; Maroon (long dashed): male.

In 2016, the proportion of males with heavy-intensity infections was higher than that of females (*p* = 0.013) (Table B in S1 Text). The age distribution of *S*. *mansoni* infection prevalence and intensity is shown in Fig 6. The highest egg counts were found among boys 10–14 years (14,424 epg) and the differences in the intensity of infection across the eight age groups were highly significant for both sexes (*p*<0.001).

In 2017, the proportion of males with heavy-intensity infections was lower than that of females, but not significant (*p* = 0.564) (Table F in S1 Text). The age distribution of *S*. *mansoni* infection prevalence and intensity is shown in Fig 6 and Table G in S1 Text, respectively. The differences in the intensity of infection across the eight age groups were the same as in 2016, highly significant for both sexes (*p*<0.001) and corresponding to the highs and lows of infection prevalence, as described above.

Fig 5 shows a map of Ituri province with the *S*. *mansoni* village prevalence rates observed in the 2016 and 2017 surveys. The rates were generally higher in the South than in the North and were highest in the lowlands and along the shores of Lake Albert.

### *Schistosoma mansoni* infection prevalence and intensity by age and sex

The relationship between *S*. *mansoni* infection prevalence and intensity and age is displayed in Fig 6. In both the 2016 and 2017 studies, prevalence and intensity increased proportionally with age among both female and male participants until max. 29 years. *S*. *mansoni* infection prevalence peaked at around 50.0% in 2016, and at around 80.0% in 2017. While prevalence was lower in 2016, peak intensity was higher (207.3 epg) in 2016, and lower (104.1 epg) in 2017. Prevalence was highest among children aged 15–19 years in 2016 and decreased among older participants. Prevalence was highest among participants aged 10–14 years in 2017. Overall, the male curves peaked higher in prevalence rates in males than in females in both 2016 and in 2017. Whereas infection intensity was higher among males than among females in 2016, it was similar among males and females in 2017. The trends for different age groups, in terms of prevalence and intensity, are almost the same. However, a slight difference is observed in 2017: while infection intensity decreased abruptly for males, it remained a plateau for females.

### Correlation between the *Schistosoma mansoni* infection prevalence and intensity

**Fig 7:**
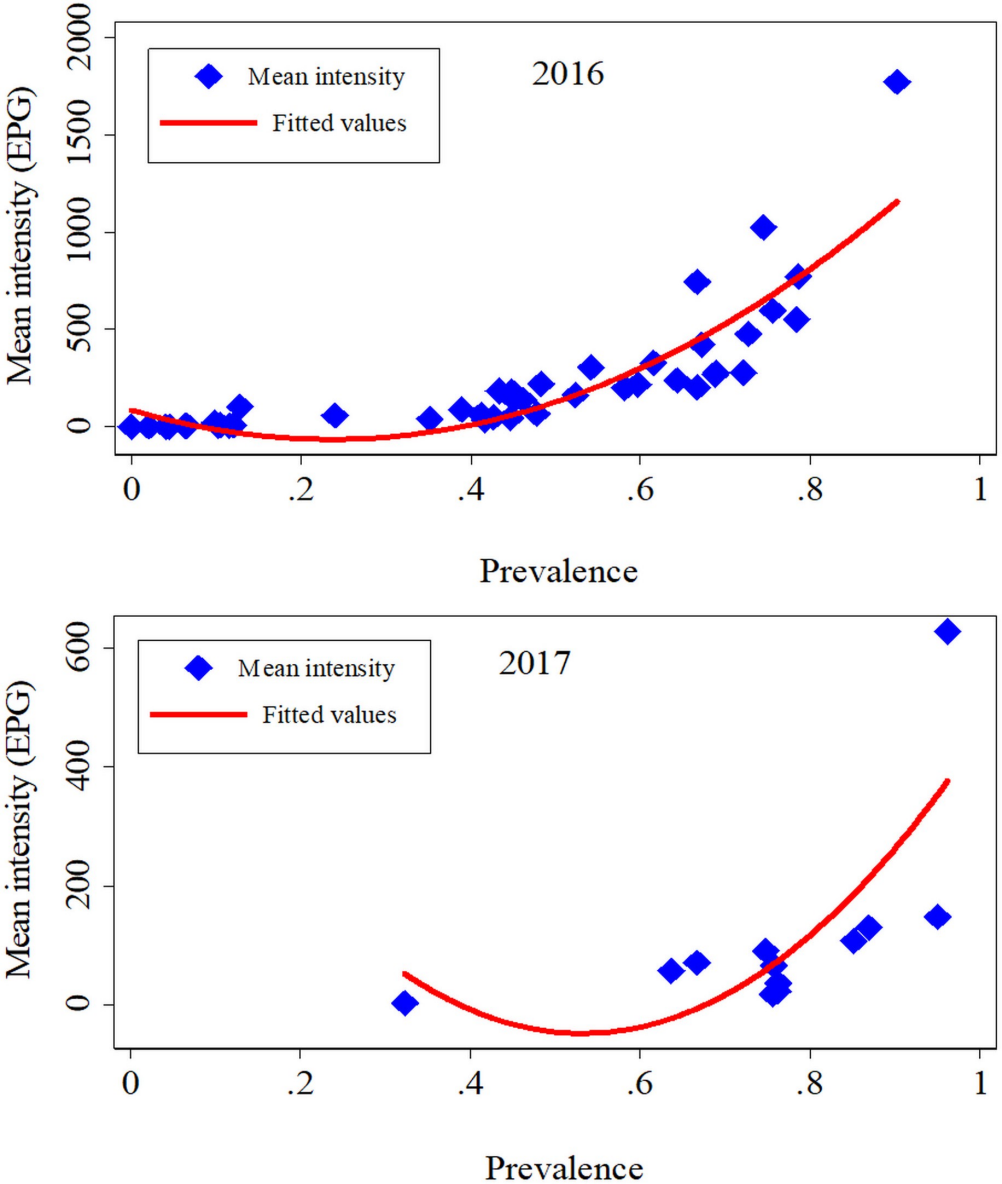
**Correlation between *Schistosoma mansoni* infection prevalence and intensity at village level in the 2016 geographical study (top) and the 2017 in–depth study (bottom).** All quadratic line fitted values.

The relationship between *S*. *mansoni* infection prevalence and intensity is displayed in Fig 7. *S*. *mansoni* village–level infection prevalence was positively correlated with infection intensity in the 2016 and 2017 studies.

**Fig 8:**
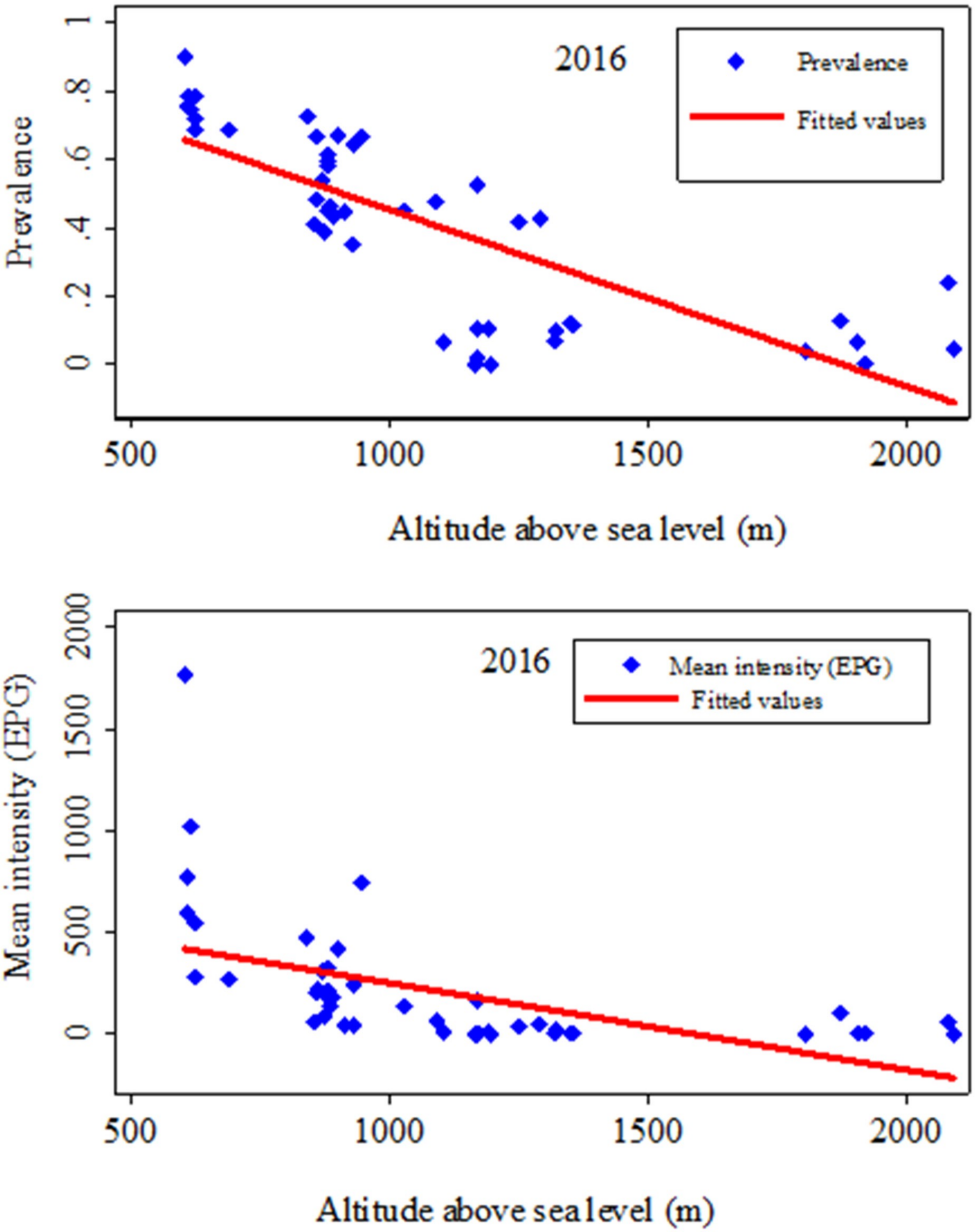
Correlation of *S*. *mansoni* infection prevalence and intensity with altitude (meters above sea level) in the 2016 distribution study. The figure above shows the influence of altitude on *S*. *mansoni* infection prevalence (top) and intensity expressed as an arithmetic mean (bottom) as observed in 2016. The higher the altitude, the lower the infection prevalence and intensity.

A relationship was also observed between *S*. *mansoni* village–level infection prevalence and intensity and altitude in the 2016 study (Fig 8). The *S*. *mansoni* infection prevalence and intensity were negatively correlated with altitude.

### Risk factors for *Schistosoma mansoni* infection

Tables N–Q in S1 Text show the results of the univariable risk analysis for an *S*. *mansoni* infection with demographic, socioeconomic, environmental, behavioural, family, and individual variables. A significant increase in risk is associated with age (10–29 years), ethnicity (Sudanese), duration of residence (≥10 years), bad housing, and not owning shoes. *S*. *mansoni* infection was also associated with living in the health districts of Bunia, Tchomia, Angumu, Lolwa and Mandima, and with proximity of the household to a nearby body of water. The absence of a latrine in the household, washing clothing in streams, and farming were among the most high-risk behaviours. Knowledge about the use of praziquantel was found to be a protective factor against *S*. *mansoni* infection.

Predictive variables with a significance level of less than 20% in the univariate model were retained in the multivariate risk factor model. Gender was also included in the multivariate logistic regression model (Table R in S1 Text).

**Fig 9:**
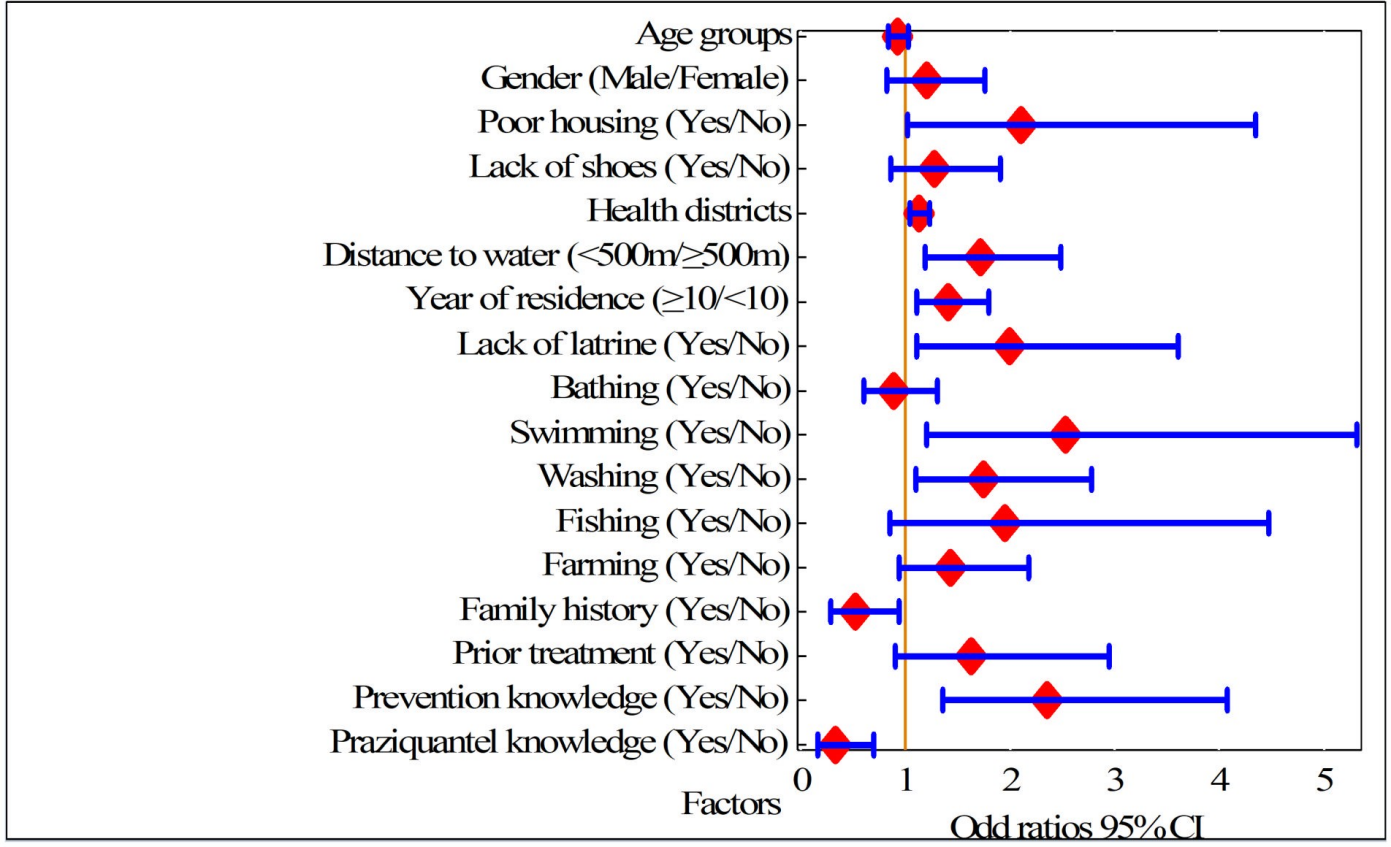
Results of multivariable logistic regression analysis for *S*. *mansoni* infection in 2017. Risk analysis performed for participants from 12 villages in Ituri province (n = 707). Adjusted Odds ratios, large red diamond and 95% confidence interval (CI), range indicated by horizontal blue lines.

Fig 9 presents the results of the multivariate risk factor analysis. Ten of 17 variables were significantly associated with *S*. *mansoni* infection. Participants living in certain health districts, such as Bunia, the unique urban area; Tchomia and Angumu, at the shore of Lake Albert; and Lolwa and Mandima, at the southern and forest–covered region of the province (aOR 1.13, 95% CI 1.04–1.23, p<0.005) had a significantly increased infection risk. Those with poorly built households (aOR 2.10, 95% CI 1.02–4.35, p<0.044), or without a latrine (aOR 2.00, 95% CI 1.11–3.60, p = 0.022) had higher odds of being infected. Water contact activities such as swimming (aOR 2.53, 95% CI 1.20–5.32, p = 0.014) and washing clothes in streams (aOR 1.75, 95% CI 1.10–2.78, p = 0.018) were also associated with a significant risk increase. Furthermore, infection risk increased significantly with a longer residence period (≥10 years *vs* shorter period: aOR 1.41, 95% CI 1.11–1.79, p = 0.005) and closer proximity to water bodies (aOR 1.72, 95% CI 1.18–2.49, p = 0.005). Finally, participants who had a family history of schistosomiasis (aOR 0.52, 95% CI 0.29–0.94, p = 0.030) and those with knowledge of praziquantel treatment had a significantly lower *S*. *mansoni* infection risk (aOR 0.33, 95% CI 0.16–0.69, p = 0.003).

## Discussion

Intestinal schistosomiasis is one of the major neglected tropical and poverty–related diseases. *S*. *mansoni* thrives in tropical and sub–tropical regions with poor sanitation conditions [1, 4, 46] and threatens about 393 million people in Africa, the Middle East, Brazil, Venezuela, Suriname, and the Caribbean. About 54 million people are infected in these regions [47]. Children bear the highest burden of infection due to inadequate hygiene and their frequent contact with infected waters [48].

To the best of our knowledge, this study is the first comprehensive, province–wide assessment of *S*. *mansoni* infection prevalence, intensity, and risk factors in Ituri since colonial times. In two studies, encompassing 14 (38.9%) of the 36 health districts in Ituri province, including 56 villages and more than 2,800 participants, we found a very high *S*. *mansoni* infection burden, revealing a major public health problem in the province. At the outset (2016), we used the Kato-Katz test alone and, alarmed by the findings, subsequently combined Kato-Katz and POC-CCA tests results to achieve a more sensitive diagnostic approach. The *S*. *mansoni* infection prevalence levels were higher in 2017 when the diagnostic approach combined the results of two stool samples examined by Kato-Katz and CCA in urine was used. This observation raises the issue of the sensitivity of this standard technique [49].

In the 2016 study, conducted in 46 villages across 12 health districts, 40.0% of the study participants tested positive for *S*. *mansoni*. Infection prevalence and infection intensity showed similar trends among men and women, though they were slightly higher for males than for females. We also found that *S*. *mansoni* infection had evidently been acquired early in life, as many children under five years were infected. Both prevalence and intensity peaked among those 10–14 and 15–19 years old. These results likely reflect men’s frequent exposure to water through fishing and farming activities. Culturally, in Ituri, men are responsible for meeting the food, clothing, and financial needs of their families, while women tend to domestic chores such as fetching water, washing clothes and dishes, caring for children, and preparing food. Thus, women have less contact with contaminated water than men. In endemic areas, children in particular spend long hours swimming, playing, bathing, or fetching water from water bodies that may contain cercariae. They also defaecate indiscriminately in the surrounding environment, causing most of the contamination and increasing their own risk of infection [50].

Considerable variability in infection prevalence was observed at the health district level, ranging from 3.9% in Adi, to 80.2% in Nyarambe. Three villages were free of *S*. *mansoni* infection, two in the north and one in the high hill region. We cannot account for this finding in the two northern villages, as they are both built near streams that are currently used for washing and bathing. However, our results are consistent with the low *S*. *mansoni* infection rate reported in colonial times, when prevalence in Aru territory was found to be below 3.0% [9, 51]. Fundi’s high altitude, cooler temperature and water velocity is not suitable for snail reproduction. For the same reasons, people may also have less contact with water, thereby reducing the probability of infection [52, 53]. In general, we observed higher infection rates in the south and east of the province compared to the north and west. Data from colonial times [54, 55] and from the mid–1980s [56] showed a similar geographical pattern. Indeed, exposure to the waters of Lake Albert explain this distribution pattern to a large extent. *Biomphalaria* molluscs are abundant in the lake’s shallow waters and inhabitants of the lake–side villages intensively use the water for various activities. We also found a negative association between *S*. *mansoni* infection and altitude. In the colder hillside areas, *Biomphalaria* mollusc development decelerates, and human water contact is less frequent, leading to reduced transmission of *S*. *mansoni* infection [52]. Hence, the Blue Mountains chain of Ituri contributes to a lower infection prevalence pattern in the east of the province.

Surprisingly, *S*. *mansoni* infection prevalence was very high in the villages situated in the southwestern forest–covered part of the province, i.e. the prevalence in Lolwa and Mandima health districts exceeded the rates recorded at the lakeshore. However, this region is situated in the lowlands and the population depends on the existing water bodies in the area.

We observed that prevalence was slightly higher in rural areas compared to urban areas. Schistosomiasis particularly affects poor communities [1, 57–59] without the means to protect themselves from risky water contacts. Hence, rural communities are especially affected [60–64], but suburban and urban poor communities should also be considered [65–67]. Thus, it is not surprising that the participants living in Ngezi village in Bunia city had a very high *S*. *mansoni* infection burden, comparable to villages on the shore of Lake Albert. In fact, Ngezi village is situated along the Nyamukau and Ngezi rivers, where adequate water supply and sanitation is lacking.

Infection prevalence was lowest in the health district of Nia–Nia, in the southwestern corner of the province. The remoteness of the villages and the scarcity of water explain the low infection rate. Furthermore, the intermediate host snail *Biomphalaria alexandrina stanleyi* described in this region [68] is thought to be less effective in transmitting *S*. *mansoni*.

We would expect the infection intensity to show a similar geographical distribution pattern as the infection prevalence. Indeed, in both the 2016 and 2017 studies, we observed a positive association between infection prevalence and intensity at the village level. Similar observations have been made in other endemic settings for *S*. *mansoni* [60] and other *Schistosoma* species [69]. In general, variable transmission intensity is thought to account for these observations [70].

In 2017, we observed an overall infection prevalence of *S*. *mansoni* infection of almost double of 2016. Certainly, the different sampling procedure might account for the higher prevalence rate, as we purposely focused on known *S*. *mansoni* endemic villages. However, in 2017 we also employed a more sensitive diagnostic approach, consisting of an examination of two stools (4 Kato–Katz smears) and a urine sample (POC–CCA rapid test) per study participant. The infection prevalence ranged from 52.7% to 95.0% at health district level and from 32.3% to 96.2% at village level.

The Kato–Katz technique remains the standard method for diagnosing *S*. *mansoni*, however it has some shortcomings. It has a low sensitivity; it is time consuming, and it requires skilled and trained technicians to identify the *S*. *mansoni* eggs microscopically. In addition, its sensitivity decreases with decreasing infection intensity [71]. The recently developed POC–CCA test [44] offers an alternative method. Its use in our study increased the number of *S*. *mansoni* patients identified. Of the 707 participants screened for *S*. *mansoni* using both techniques, the prevalence high, varying from 55.0% using the Kato–Katz technique and 73.1% when using combined diagnostic approach. These observations are corroborated by the results reported by Okoyo and colleagues [72] when comparing the performance of CCA and KK techniques for evaluating *S*. *mansoni* infection in areas with low prevalence in Kenya. They found that using the CCA technique increased diagnostic accuracy. There is some discrepancy when comparing our results with those of Standley and colleagues [73], who found that CCA and KK techniques had a similar degree of accuracy. Recent publications discuss the specificity of POC–CCA. POC–CCA showed some cross–reactivity with intestinal nematodes and other health conditions, and therefore might overestimate the prevalence [74, 75]. New diagnostic test modalities on the horizon may contribute to improving our understanding of the relative values of different diagnostic tests [76].

The 2017 in–depth study was primarily conducted to assess the most important risk factors for an *S*. *mansoni* infection. We examined the demographic, socioeconomic, environmental, behavioural and knowledge risk factors. Among the most important risk factors, our multivariable logistic regression analysis identified socioeconomic factors, such as poor housing; environmental factors, such as living in a risky health districts, in close proximity to water bodies for long time periods; behavioural factors, such as the lack of a latrine, and swimming and washing in waterbodies; and knowledge factors [50].

In our study, gender was not a risk factor for infection as females and males had similar levels of infection prevalence in both studies, with a difference <2.0% in 2017 and >5.0% in 2016. These results are consistent with those of other authors [77, 78]. In Ituri province, men and women have different types of water contact activities, but contact intensities are comparable.

Age was an important risk factor. *S*. *mansoni* infection prevalence and intensity follow a typical age peak curve. In both studies, the prevalence and intensity increased with age until it peaked among the groups aged 10–19 years. Interestingly, the infection prevalence peak was higher in 2017 (86.1% *vs* 50.2% in 2016), but infection intensity was lower that year (45 epg *vs* 245 epg in 2016). Indeed, the use of a higher sensitivity diagnostic approach in 2017 is mainly responsible for these observations. Children in these age groups are excessively mobile and are exposed to infected water while swimming, playing, bathing, washing clothes, or fetching water [79]. Our results resemble the patterns reported by Kabatereine and colleagues [80] when describing the epidemiology of *S*. *mansoni* infections on the Ugandan side of Lake Albert, and by Tukahebwa and colleagues [77] when investigating *S*. *mansoni* infection in a fishing community on the shores of Lake Victoria.

A striking finding of our study is that children under five years are highly infected with *S*. *mansoni* (62.3% in 2017). This demonstrates the early life exposure of young children through bathing and playing in infested waters. A similar finding was reported by Nalugwa and colleagues [81], who described the high *S*. *mansoni* infection prevalence among preschool children in communities along Lake Victoria in Uganda.

Beyond the scope, we also did an investigation of *S*. *mansoni* and *S*. *haematobium* among 200 motorcycle and car cleaners in Bunia city in 2016 using both Kato-Katz and urine microscopy and strip tests (Combina 10 M, Human Diagnostics Uganda), but did not use POC-CCA tests. However, even prevalence of *S*. *mansoni* was very high, we did not find any case of *S*. *haematobium*.

Our study has some limitations. First, the two studies used different sampling techniques. In 2016, our sample came from those first to arrive in the centre of the village, thereby introducing a possible selection bias. Likewise, the population, especially in the rural areas, is generally averse to participating in research endeavours, adhering more readily to interventions than to preliminary investigations. Thus, the study compliance in some villages was quite low. Men, in particular, adhered less to the procedures. This difficulty resulted in fewer study participants in some villages. Second, the two studies used different diagnostic approaches. The POC-CCA test was partially used in 2017, and molecular tests such as polymerase chain reaction (PCR) and/or loop-mediated isothermal amplification (LAMP) were not available in the region at that time. Due to the prevailing insecurity due to armed groups in the province in 2016, we could only spend a maximum of two days in each village. Therefore, only one stool sample could be collected from each study participant. Given the low sensitivity of the diagnostic approach employed in 2016, we underestimated the true infection rates. Third, the risk factor analysis should be interpreted with caution, as the in–depth study did not include health districts and villages from the northern or hilly regions. Hence, some relevant risk factors might be missing. Fourth, a concurrent malacological survey to assess the prevalence and rates of cercarial infection among snails in the region could have revealed valuable information regarding disease transmission and its seasonality, which we have not considered here.

The DRC’s national NTD control programme succeeded in mapping schistosomiasis and STH in 99.2% of the country’s health districts in 2015. It subsequently launched a master plan for 2016–2020 to work toward eliminating schistosomiasis as a public health problem by 2025, following the WHO guidelines. These guidelines depend on the level of infection as assessed among school-age children (SAC). The ensuing recommendations state that preventive-chemotherapy (PCT) mass drug administration (MDA) should take place as follows: i) annually for SAC and high-risk adults (HRA) in highly-endemic communities (prevalence >50.0% in SAC); ii) every-other-year for SAC and selected high-risk adults in moderate- to high-prevalence communities (10–49.0%); and iii) twice for SAC during their primary school years in communities where prevalence is low (<10.0% in SAC) [5]. Another objective of the DRC’s NTD programme is to interrupt transmission by increasing access to adequate sanitation and drinking water and by improving the immediate environment of communities [33]. In Ituri province, control activities are performed by the provincial NTD branches situated in Aru and Bunia, which implement the control activities in the northern and southern health districts, respectively. To the best of our knowledge, only three health districts along the shores of Lake Albert, namely Tchomia, Angumu, and Nyarambe, benefit from the first strategy, while all the others either use the strategies for communities with moderate and low prevalence or have not yet started PCT. As the country is vast and without adequate roads or trains, supplying medicines is a major challenge. Health districts routinely obtain their supplies from neighbouring Uganda. People, especially children, make greater use of water courses for domestic, recreational, and occupational activities, which exposes them to infection. Our data will be of practical value to help improve control interventions, especially as COVID-19 became currently a huge global problem. It is likely that this virus will make the work to be done more complicated, not to mention the additional disease burden for the people.

The results of this study are important in several respects. They show that (i) *S*. *mansoni* is highly prevalent in Ituri province. The infection prevalences observed are beyond those reported in the 1960s [55] and those described in other parts of the country [11, 13, 18, 21, 22]. The observed infection levels are comparable with those in Ugandan fishing villages [77, 78]. The results also show that (ii) the age distribution of infection prevalence and intensity follows the typical pattern of an *S*. *mansoni* endemic area, where control measures are insufficiently implemented; and that (iii) preschool children bear a high infection burden and therefore deserve special attention in the control programme.

In conclusion, our results provide comprehensive baseline data showing that *S*. *mansoni* is highly endemic and is a major health concern in Ituri province, DRC. Infection prevalence and intensity, and its relationship with the prevailing socioeconomic, environmental, and behavioural risk factors reflect intense exposure and alarming transmission rates. Our findings call for a more robust plan of action for controlling and, eventually, eliminating *S*. *mansoni* infection in Ituri province. Intervention strategies could include full implementation of the WHO recommendations for mass drug administration (MDA), by treating communities adequately and according to their real needs. Additional efforts are required to strengthen and expand community-based programmes that promote practices aimed at preventing the spread of *S*. *mansoni* and other parasitic infections. Comprehensive community-based health education, and implementation of water, hygiene, and sanitation (WASH) programmes in both rural and urban areas are of high value, especially in the context of the current COVID-19 pandemic, which further complicates the work to be done and adds to the country’s mortality and morbidity burden. Combined, these efforts are likely to yield appreciable and sustainable gains towards improving public health and welfare in Ituri province.

## Supporting information

S1 Text **Table A in S1 Text. *Schistosoma mansoni* infection prevalence and intensity, global results of the two: 2016 and 2017 studies.** Results obtained with 1KK in 2016 (n = 2,131) and 1KK, 2KK, one POC-CCA, and 2KK+POC-CCA in 2017 (n = 707). **Table B in S1 Text**. ***Schistosoma mansoni* infection prevalence and intensity in the 2016 geographical distribution study.** Study conducted in 46 villages in Ituri province (n = 2,131). One stool sample from each study participant was examined with the Kato–Katz test (two smears per stool). **Table C in S1 Text**. ***Schistosoma mansoni* infection prevalence and intensity in the 2016 geographical distribution study by gender.** Study conducted in 46 villages in Ituri province (n = 2,131). One stool sample from each study participant was examined with the Kato–Katz test (two smears per stool) by sex among different age groups, residence, ethnic group, and altitude. **Table D in S1 Text**. ***Schistosoma mansoni* infection prevalence and intensity in the 2016 geographical distribution study.** Study conducted in 46 villages in Ituri province (n = 2,131). One stool sample from each study participant was examined with the Kato–Katz test (two smears per stool). **Table E in S1 Text**. ***Schistosoma mansoni* infection prevalence and intensity in the 2017 in–depth study.** Study conducted in 12 purposively selected villages in Ituri province (n = 707). Study participants provided two stool samples. From each sample, two Kato–Katz (KK) smears were examined (total four smears per person). In addition, each participant provided a urine sample for point–of–care circulating cathodic antigen (POC–CCA) test. Kato-Katz + POC-CCA combined results. **Table F in S1_Text**. ***Schistosoma mansoni* infection prevalence and intensity in the 2017 in–depth study.** Study conducted in 12 purposively selected villages in Ituri province (n = 707). Study participants provided two stool samples. From each sample, when one Kato–Katz (KK) smear was examined (total two smears per person). KK overall results taken in account alone.by sex, age categories, residence, ethnic groups, and altitude. **Table G in S1_Text**. ***Schistosoma mansoni* infection prevalence and intensity in the 2017 in–depth study.** Study conducted in 12 purposively selected villages in Ituri province (n = 707). Study participants provided two stool samples. From each sample, two Kato–Katz (KK) smears were examined (total four smears per person). In addition, each participant provided a urine sample for point–of–care circulating cathodic antigen (POC–CCA) test. POC-CCA results alone (for prevalence). **Table H in S1 Text**. ***Schistosoma mansoni* infection prevalence and intensity in the 2017 in–depth study.** Study conducted in 12 purposively selected villages in Ituri province (n = 707). Study participants provided two stool samples. From each sample, two Kato–Katz (KK) smears were examined (total four smears per person). KK overall results taken in account alone.by sex, age categories, residence, ethnic groups, and altitude. **Table I in S1 Text**. ***Schistosoma mansoni* infection prevalence and intensity in the 2017 in–depth study.** Study conducted in 12 purposively selected villages in Ituri province (n = 707). Study participants provided two stool samples. From each sample, two Kato–Katz (KK) smears were examined (total four smears per person). KK results taken in account alone.by sex, age categories, residence, ethnic groups, and altitude. **Table J in S1 Text**. ***Schistosoma mansoni* infection prevalence in the 2017 in-depth study.** Study conducted in 12 villages in Ituri province (n = 707). From each sample, two Kato–Katz (KK) smears were examined (total four smears per person). In addition, each participant provided a urine sample for point–of–care circulating cathodic antigen (POC–CCA) test. Results of the 4 different diagnostic approaches (1KK, 2KK, POC-CCA, and combined 2KK+POC-CCA). **Table K in S1 Text**. ***Schistosoma mansoni* infection prevalence and intensity in the 2017 in–depth study.** Study conducted in 12 purposively selected villages in Ituri province (n = 707). Study participants provided two stool samples. From each sample, two Kato–Katz (KK) smears were examined (total four smears per person). In addition, each participant provided a urine sample for point–of–care circulating cathodic antigen (POC–CCA) test. Combined KK+POC-CCA results. **Table L in S1 Text**. ***Schistosoma mansoni* infection prevalence and intensity in the 2017 in–depth study.** Study conducted in 12 purposively selected villages in Ituri province (n = 707). Study participants provided two stool samples. From each sample, two Kato–Katz (KK) smears were examined (total four smears per person). In addition, each participant provided a urine sample for point–of–care circulating cathodic antigen (POC–CCA) test. Only POC-CCA results. **Table M in S1 Text**. ***Schistosoma mansoni* infection prevalence (%) in the 2017 in–depth study.** Study conducted in 12 purposively selected villages in Ituri province (n = 707). Study participants provided two stool samples. From each sample, one to two Kato–Katz (KK) smears were examined (total two to four smears per person). In addition, each participant provided a urine sample for point–of–care circulating cathodic antigen (POC–CCA) test. POC-CCA results. Prevalence of the three diagnostic approaches by sex. **Table N in S1_Text**. **Demographic and socioeconomic risk factors for *Schistosoma mansoni* infection (2017 in–depth study).** Results obtained with the univariate analysis of risk groups for infection with *S*. *mansoni* among participants from 12 villages in Ituri province in 2017 (n = 707). **Table O in S1 Text**. **Environmental risk factors for *Schistosoma mansoni* infection (2017 in–depth study).** Results of the univariate analysis of risk groups for infection with *S*. *mansoni* among participants from 12 villages in Ituri province (n = 707). **Table P in S1 Text**. **Behavioural risk factors for *Schistosoma mansoni* infection (2017 in–depth study).** Results of the univariate analysis of risk groups for infection with *Schistosoma mansoni* among participants from 12 villages in Ituri province (n = 707). **Table Q in S1 Text**. **Family and individual risk factors for *Schistosoma mansoni* infection (2017 in–depth study).** Results of the univariate analysis of risk groups for infection with *Schistosoma mansoni* among participants from 12 villages in Ituri province (n = 707). **Table R in S1 Text**. **Risk factors for *Schistosoma mansoni* infection (2017 in–depth study).** Results of the multivariable analysis of risk groups for infection with *Schistosoma mansoni* among participants from 12 villages in Ituri province (n = 707).
(DOCX)
