# Supplementary material for: Epidemiology of Schistosoma mansoni infection in Ituri Province, north-eastern Democratic Republic of the Congo
Source: PLoS Negl Trop Dis. 2021 Dec 2;15(12):e0009486. doi: 10.1371/journal.pntd.0009486 (PMC8638996; doi:10.1371/journal.pntd.0009486)
Supplement: S1 Text — Table A in S1 Text. Schistosoma mansoni infection prevalence and intensity, global results of the two: 2016 and 2017 studies. Results obtained with 1KK in 2016 (n = 2,131) and 1KK, 2KK, one POC-CCA, and 2KK+POC-CCA in 2017 (n = 707). Table B in S1 Text. Schistosoma mansoni infection prevalence and intensity in the 2016 geographical distribution study. Study conducted in 46 villages in Ituri province (n = 2,131). One stool sample from each study participant was examined with the Kato–Katz test (two smears per stool). Table C in S1 Text. Schistosoma mansoni infection prevalence and intensity in the 2016 geographical distribution study by gender. Study conducted in 46 villages in Ituri province (n = 2,131). One stool sample from each study participant was examined with the Kato–Katz test (two smears per stool) by sex among different age groups, residence, ethnic group, and altitude. Table D in S1 Text. Schistosoma mansoni infection prevalence and intensity in the 2016 geographical distribution study. Study conducted in 46 villages in Ituri province (n = 2,131). One stool sample from each study participant was examined with the Kato–Katz test (two smears per stool). Table E in S1 Text. Schistosoma mansoni infection prevalence and intensity in the 2017 in–depth study. Study conducted in 12 purposively selected villages in Ituri province (n = 707). Study participants provided two stool samples. From each sample, two Kato–Katz (KK) smears were examined (total four smears per person). In addition, each participant provided a urine sample for point–of–care circulating cathodic antigen (POC–CCA) test. Kato-Katz + POC-CCA combined results. Table F in S1_Text. Schistosoma mansoni infection prevalence and intensity in the 2017 in–depth study. Study conducted in 12 purposively selected villages in Ituri province (n = 707). Study participants provided two stool samples. From each sample, when one Kato–Katz (KK) smear was examined (total two smears per person). KK overall results t [file pntd.0009486.s001.docx]

**S1 Text**

**Table A. *Schistosoma mansoni* infection prevalence and intensity, global results of the two: 2016 and 2017 studies.** Results obtained with 1KK in 2016 (n=2,131) and 1KK, 2KK, one POC-CCA, and 2KK+POC-CCA in 2017 (n=707).

Year/Test Total Infected Prevalence (95% CI) EPG (95% CI)

N n

2016

1KK (2 smears) 2,131 852 40.0 (37.9–42.1) 207.4 (173.3-241.5)

2017

1KK (2 smears) 707 272 38.5 (34.9–42.1) 100.9 (72.0-129.7)

2KK (4 smears) 707 389 55.0 (51.3–58.7) 104.1 (77.1-131.1)

One POC-CCA 707 442 62.5 (58.9–66.1) NA

2KK+POC-CCA 707 517 73.1 (69.9–76.4) 104.1 (77.1-131.1)

EPG = eggs per gram; KK= Kato-Katz test (1KK – one stool sample – two smears and 2KK – two stool samples – four smears). POC-CCA = Point-of-care circulating cathodic antigen urine test. 2KK+POC-CCA = approach of combining 2KK with one POC-CCA. NA = not applicable.

**Table B. *Schistosoma mansoni* infection prevalence and intensity in the 2016 geographical distribution study.** Study conducted in 46 villages in Ituri province (n=2,131). One stool sample from each study participant was examined with the Kato–Katz test (two smears per stool).

Characteristic Prevalence (%) Infection Intensity (%) EPG (Arithmetic)

_______ ________________________________

Positive Light Moderate Heavy Mean Maximum

Overall 40.0 17.2 11.1 11.7 207.4 14,424

Sex

Female 37.4 17.2 10.6 9.7 171.6 10,680

Male 42.6 17.2 11.7 13.8 244.6 14,424

*p*-value 0.015 0.013

Age group (years)

1 – 4 7.7 3.9 3.9 0 17.5 360

5 – 9 41.3 18.6 11.5 11.2 213.9 10,680

10 – 14 45.0 17.9 11.3 15.7 313.5 14,424

15 – 19 50.2 14.1 15.4 20.8 330.1 13,248

20 – 29 46.6 21.1 13.1 12.5 187.4 3,312

30 – 39 40.3 18.6 15.3 6.4 144.6 7,824

40 – 49 26.1 13.6 5.5 7.0 70.3 1,296

**≥**50 13.8 11.9 1.3 0.6 11.7 744

*p*-value <0.001 <0.001

Residence

Rural 40.5 16.6 11.2 12.7 223.4 14,424

Urban 33.9 23.3 10.0 0.6 33.2 624

*p*-value 0.081 <0.001

Ethnic group

Nilo-Hamite 47.2 26.4 10.4 10.4 141.7 3,096

Bantu 48.0 24.6 15.1 8.3 143.5 9,792

Nilotic 47.4 15.0 12.9 19.5 376.0 14,424

Sudan* 18.4 11.0 4.3 3.1 43.0 5,136

Pygmy 50.3 21.6 15.1 13.7 211.1 7,824

*p*-value <0.001 <0.001

Altitude

<1000 m 61.2 22.4 17.4 21.4 377.8 14,424

1000–1800 m 22.7 14.5 6.0 2.2 41.8 4,080

>1800 m 8.7 5.2 1.7 1.7 27.8 3,312

*p*-value <0.001 <0.001

EPG = eggs per gram; Mean = mean number of epg; Maximum = maximum number of epg; *p*-value = probability value of comparing infection prevalence and heavy infection intensity by sex, age groups, residence, ethnic groups, and altitude.

**Table C:** ***Schistosoma mansoni* infection prevalence and intensity in the 2016 geographical distribution study by gender.** Study conducted in 46 villages in Ituri province (n=2,131). One stool sample from each study participant was examined with the Kato–Katz test (two smears per stool) by sex among different age groups, residence, ethnic group, and altitude.

**Sex Female Male**

**Characteristics** Prev. Heavy Mean Max Prev. Heavy Mean Max

Overall 37.4 9.6 171.6 10,680 42.6 13.8 244.6 14,424

Age group (years)

1 – 4 8.3 0 8.0 96 7.1 0 25.7 360

5 – 9 43.1 12.9 271.6 10,680 39.7 9.8 164.1 6,672

10 – 14 43.4 11.4 225.5 8,376 46.3 19.4 388.3 14,424

15 – 19 45.8 14.4 195.7 3,336 54.5 26.8 459.1 13,248

20 – 29 42.3 12.0 160.0 3,096 53.5 13.2 231.6 3,312

30 – 39 37.1 5.3 142.5 7,824 44.2 7.7 147.1 2,760

40 – 49 16.5 2.6 33.2 984 39.3 13.1 121.1 1,296

**≥**50 13.9 1.4 15.7 744 13.6 0 8.5 144

*p*-value <0.001 <0.001 <0.001 <0.001

Residence

Rural 37.7 10.5 184.3 10,680 43.5 15.0 264.0 14,424

Urban 34.7 1.1 39.2 624 32.9 0 26.5 288

*p*-value 0.568 0.017 0.060 0.001

Ethnic group

Nilo-Hamite 39.6 12.5 162.0 3,096 53.5 8.6 125.0 1,344

Bantu 46.0 6.3 109.9 4,080 50.6 10.8 185.7 9,792

Nilotic 46.2 16.6 318.8 10,680 48.6 22.3 430.6 14,424

Sudanese 15.4 1.7 23.7 864 21.5 4.5 62.6 5,136

Pygmy 45.5 12.2 192.6 7,824 55.9 15.4 232.3 2,688

*p*-value <0.001 <0.001 <0.001 <0.001

Altitude

<1000 m 57.4 17.3 307.4 10,680 65.6 25.6 451.2 14,424

1000-1800 m 22.4 2.5 46.3 4,080 23.0 1.9 37.0 2,088

**>**1800 m 5.6 0.7 9.0 744 11.7 2.8 46.3 3,312

*p*-value <0.001 <0.001 <0.001 <0.001

Prev. = infection prevalence; Heavy = prevalence of heavy infection intensity; Mean = mean arithmetic egg per gram (epg); Max = maximum egg per gram (epg); *p* -value = probability value comparing infection prevalence and heavy infection intensity among females and males by age groups, residence, ethnic groups, and altitude.

**Table D: *Schistosoma mansoni* infection prevalence and intensity in the 2016 geographical distribution study.** Study conducted in 46 villages in Ituri province (n=2,131). One stool sample from each study participant was examined with the Kato–Katz test (two smears per stool) by village and health district.

**Entity Prevalence Intensity (%) EPG (Arithmetic)**

Total n (%) Light Mod. Heavy Mean Max

**Villages**

Overall 2,131 852 (40.0) 17.2 11.1 11.7 207.4 14,424

Ombanya 48 0 (0) 0 0 0 0 0

Upepeni 39 0 (0) 0 0 0 0 0

Fundi 49 0 (0) 0 0 0 0 0

Moze 50 1 (2.0) 2.0 0 0 0.5 24

Upani-Logo 50 2 (4.0) 4.0 0 0 1.0 24

Nioka-Foret 45 2 (4.4) 4.4 0 0 1.1 24

Aubha 47 3 (6.4) 4.3 2.1 0 5.6 120

Kpana 47 3 (6.4) 6.4 0 0 2.0 48

Nyaradha 46 3 (6.5) 4.4 2.2 0 5.2 144

Duba 51 5 (9.8) 7.8 0 2.0 20.2 864

Laybo 49 5 (10.2) 10.2 0 0 6.4 96

Iri 38 4 (10.5) 5.3 5.3 0 10.1 192

Nyangaray 52 6 (11.5) 11.5 0 0 5.1 72

Saio 50 6 (12.0) 10.0 2.0 0 7.2 120

Umoyo 47 6 (12.8) 2.1 6.4 4.3 103.1 3,312

Ureli 50 12 (24.0) 14.0 4.0 6.0 59.0 912

Mangiva 37 13 (35.1) 18.9 16.2 0 40.2 288

Bandilesu 18 7 (38.9) 16.7 16.7 5.6 88.0 672

Lolua 51 21 (41.2) 25.5 9.8 5.9 61.2 816

Ngezi 48 20 (41.7) 31.3 10.4 0 35.0 240

Yambi 82 35 (42.7) 26.8 14.6 1.2 48.0 624

Bolombo 90 39 (43.3) 21.1 13.3 8.9 184.7 7,824

Makwange 47 21 (44.7) 31.9 10.6 2.1 46.0 696

Pinzili I 29 13 (44.8) 24.1 10.3 10.3 181.2 2,592

Ndenge 51 23 (45.1) 19.6 11.8 13.7 138.4 2,088

Makayanga 39 18 (46.2) 28.2 10.3 7.7 140.3 1,872

Loyi-Batine 44 21 (47.7) 27.3 15.9 4.6 66.5 768

Mungamba 29 14 (48.3) 17.2 13.8 17.2 220.1 1,584

Singoma 86 45 (52.3) 31.4 14.0 7.0 164.7 4,080

Bandibiso 24 13 (54.2) 16.7 16.7 20.8 306.0 1,752

Shaurimoya 43 25 (58.1) 16.3 30.2 11.6 199.8 2,520

Tindo 57 34 (59.7) 36.8 12.3 10.5 218.5 5,136

Pinzili II 26 16 (61.5) 30.8 7.7 23.1 327.7 2,688

Paluo 42 27 (64.3) 23.8 19.1 21.4 240.0 1,944

Ramogi 6 4 (66.7) 16.7 16.7 33.3 744.0 2,592

Takumanza 24 16 (66.7) 20.8 20.8 25.0 203.0 744

Pekele 64 43 (67.2) 14.1 25.0 28.1 421.9 9,792

Gengere 77 53 (68.8) 39.0 14.3 15.6 267.7 5,400

Kalambo 45 31 (68.9) 20.0 26.7 22.2 277.9 1,776

Pamaya 43 31 (72.1) 30.2 23.3 18.6 278.0 2,328

Jupanyarabi 55 40 (72.7) 18.2 21.8 32.7 476.5 3,576

Kalako 51 38 (74.5) 11.8 19.6 43.1 1,023.5 10,680

Ndawe 45 34 (75.6) 20.0 24.4 31.1 597.9 4,608

Mita 37 29 (78.4) 21.6 18.9 37.8 552.0 3,096

Wikidhi 42 33 (78.6) 9.5 28.6 40.5 772.6 13,248

Kolokoto 41 37 (90.2) 9.8 4.9 75.6 1,770.7 14,424

*p*-value <0.001 <0.001

**Health districts**

Overall 2,131 852 (40.0) 17.2 11.1 11.7 207.4 14,424

Adi 127 5 (3.9) 2.4 1.6 0 3.2 192

Laybo 144 8 (5.6) 4.9 0.7 0 4.0 120

Logo 144 11 (7.6) 4.2 2.1 1.4 34.7 3,312

Bambu 149 14 (9.4) 8.1 0.7 0.7 10.3 864

Rethy 144 14 (9.7) 6.3 1.4 2.1 20.8 912

Bunia 180 61 (33.9) 23.3 10.0 0.6 33.2 624

Nyankunde 181 89 (49.2) 27.1 13.8 8.9 133.4 4,080

Komanda 416 208 (50.0) 23.3 15.1 11.5 188.9 7,824

Lolwa 162 85 (52.5) 22.8 16.1 13.6 199.3 9,792

Angumu 225 158 (70.2) 26.7 19.1 24.4 392.3 5,400

Tchomia 133 98 (73.7) 17.3 21.8 34.6 640.1 10,680

Nyarambe 126 101 (80.2) 16.7 19.1 44.4 928.6 14,424

*p*-value <0.001 <0.001

Mod. = prevalence of moderate infection intensity; Heavy = prevalence of heavy infection intensity; EPG = eggs per gram; Mean = mean arithmetic egg per gram (epg); Max = maximum egg per gram (epg); *p* -value = probability value comparing infection prevalence and heavy infection intensity by villages, and health districts.

**Table E: *Schistosoma mansoni* infection prevalence and intensity in the 2017 in-depth study using one stool sample per study participant.** Study conducted in 12 purposively selected villages in Ituri province (n=707). Kato-Katz examination results of two smears per stool. Data stratified by village and health districts.

**Entity Prevalence Intensity (%) _____ EPG (Arithmetic)**

Total n (%) Light Mod. Heavy Mean Max

**Villages**

Overall 707 272 (38.5) 21.2 11.6 5.7 100.9 5,472

Bankoko 31 1 (3.2) 3.2 0 0 1.9 60

Lumumba 36 7 (19.4) 8.3 11.1 0 32.7 336

Kindia 84 18 (21.5) 15.5 6.0 0 18.3 252

Mangenengene41 9 (22.0) 22.0 0 0 8.8 72

Simbilyabo 77 23 (29.9) 18.2 7.8 3.9 70.8 1,560

Sukisa 59 23 (39.0) 30.5 8.5 0 28.9 384

Kadjugi 70 28 (40.0) 25.7 8.6 5.7 109.2 4,092

Gupe 119 49 (41.2) 21.0 13.5 6.7 83.9 1,044

Ngezi 74 35 (47.3) 25.7 16.2 5.4 83.5 1,284

Mandima 40 22 (55.0) 15.0 30.0 10.0 131.4 720

Mambau 23 14 (60.8) 39.1 13.0 8.7 150.3 1,680

Pekele 53 43 (81.1) 28.3 24.5 28.3 537.7 5,472

*p*-value <0.001 <0.001

**Health districts**

Overall 707 272 (38.5) 21.2 11.6 5.7 100.9 5,472

Nia-Nia 72 10 (13.9) 13.9 0 0 5.8 72

Tchomia 70 28 (40.0) 25.7 8.6 5.7 109.2 4,092

Angumu 119 49 (41.2) 21.0 13.5 6.7 83.9 1,044

Lolwa 76 57 (75.0) 31.6 21.0 22.4 420.5 5,472

Mandima 40 22 (55.0) 15.0 30.0 10.0 131.4 720

Bunia 330 106 (32.1) 20.3 9.7 2.1 48.6 1,560

*p*-value <0.001 <0.001

Mod. = prevalence of moderate infection intensity; Heavy = prevalence of heavy infection intensity; EPG = eggs per gram; Mean = mean arithmetic egg per gram (epg); Max = maximum egg per gram (epg); *p* -value = probability value comparing infection prevalence and heavy infection intensity by villages, and health districts.

**Table F: *Schistosoma mansoni* infection prevalence and intensity in the 2017 in–depth study using two stool samples per study participant.** Study conducted in 12 purposively selected villages in Ituri province (n=707). Study participants provided two stool samples. Kato-Katz examination results of two smears per stool on two stool samples per person (total four smears per person). Data stratified by sex, age, residence, ethnic groups, and altitude.

**Characteristics Prevalence Intensity (%) _____ EPG (Arithmetic)**

(%) Neg Light Mod. Heavy Mean Max

Overall 38.5 61.5 21.2 11.6 5.7 100.9 5,472

Sex

Female 36.2 63.8 18.6 11.8 5.8 102.5 5,472

Male 41.4 58.6 24.6 11.3 5.5 98.7 4,092

*p*-value 0.155 0.285

Age group (years)

1 – 4 19.7 80.3 14.8 4.9 0 13.2 216

5 – 9 36.1 63.9 19.0 10.8 6.3 155.7 5,472

10 – 14 50.7 49.3 25.7 16.0 9.3 122.6 1,848

15 – 19 55.9 44.1 25.0 22.1 8.8 118.9 984

20 – 29 42.2 57.8 21.7 14.5 6.0 105.0 1,680

30 – 39 33.3 66.7 20.0 8.0 5.3 80.0 2,040

40 – 49 28.6 71.4 19.6 7.1 1.8 70.7 2,868

**≥**50 25.8 74.5 21.0 3.2 1.6 24.0 408

*p*-value <0.001 0.002

Residence

Rural 44.0 56.0 36.9 14.6 9.6 146.6 5,472

Urban 32.1 67.9 34.9 10.9 2.4 48.6 1,560

*p*-value 0.001 0.001

Ethnic group

Nilo-Hamite 35.0 65.0 35.0 8.8 3.1 54.2 1,560

Bantu 37.5 62.5 36.5 13.8 5.8 100.6 5,472

Nilotic 36.7 63.3 37.4 12.1 5.4 74.5 1,308

Sudanese 55.1 44.9 31.9 20.3 17.4 273.6 4,800

*p*-value 0.027 0.017

Altitude

<1000 m 44.0 56.0 36.9 14.6 9.6 146.6 5,472

**≥**1000 m 32.1 67.9 34.9 10.9 2.4 48.6 1,560

*p*-value 0.001 0.001

Mod. = prevalence of moderate infection intensity; Heavy = prevalence of heavy infection intensity; Mean = mean arithmetic egg per gram (epg); Max = maximum egg per gram (epg); *p* -value = probability value comparing infection prevalence and heavy infection intensity by sex, age groups, residence, ethnic groups, and altitude.

**Table G: *Schistosoma mansoni* infection prevalence and intensity in the 2017 in–depth study by gender.** Study conducted in 12 purposively selected villages in Ituri province (n=707). Study participants provided two stool samples. From each sample, when one Kato–Katz (KK) smear was examined (total two smears per person). KK results taken in account alone.by sex, age categories, residence, ethnic groups, and altitude.

**Sex Female Male**

**Characteristics** Prev. Heavy Mean Max Prev. Heavy Mean Max

Overall 36.2 5.8 102.5 5,472 41.4 5.5 98.7 4,092

Age group (years)

1 – 4 13.8 0 8.3 180 25.0 0 17.6 216

5 – 9 36.4 5.2 179.1 5,472 35.8 7.4 133.5 4,092

10 – 14 48.0 8.2 105.5 816 53.5 9.9 140.1 1,848

15 – 19 50.0 12.5 132.0 732 64.3 3.6 100.3 984

20 – 29 38.8 6.0 110.9 1,680 56.3 6.3 80.3 528

30 – 39 28.6 6.1 100.2 2,040 42.3 3.9 42.0 480

40 – 49 31.3 0 17.3 156 25.0 4.2 142.0 2,868

**≥**50 22.6 3.2 29.4 408 29.0 0 18.6 120

*p*-value 0.011 0.150 0.004 0.074

Residence

Rural 40.3 9.3 149.4 5,472 49.1 8.1 142.8 4,092

Urban 31.3 1.7 47.0 1,560 33.1 2.7 50.7 1,308

*p*-value 0.064 0.008 0.004 0.018

Ethnic group

Nilo-Hamite 35.7 5.1 72.0 1,560 33.9 0 26.1 324

Bantu 34.6 4.5 98.3 5,472 41.4 5.3 103.7 4,092

Nilotic 34.1 8.0 84.1 1,044 39.7 3.9 63.7 1,308

Sudanese 51.5 9.1 265.4 4,800 58.3 19.4 281.0 2,868

*p*-value 0.293 0.702 0.122 0.006

Altitude

<1000 m 40.3 9.3 149.4 5,472 49.1 8.1 142.8 4,092

**≥**1000 m 31.3 1.7 47.0 1,560 33.1 2.7 50.7 1,308

*p*-value 0.064 0.008 0.004 0.018

Prev. = infection prevalence; Heavy = prevalence of heavy infection intensity; Mean = mean arithmetic egg per gram (epg); Max = maximum egg per gram (epg); *p* -value = probability value comparing infection prevalence and heavy infection intensity among females and males by age groups, residence, ethnic groups, and altitude.

**Table H: *Schistosoma mansoni* infection prevalence and intensity in the 2017 in–depth study.** Study conducted in 12 purposively selected villages in Ituri province (n=707). Study participants provided two stool samples. From each sample, two Kato–Katz (KK) smears were examined (total four smears per person). KK overall results taken in account alone.by sex, age categories, residence, ethnic groups, and altitude.

**Characteristics Prevalence Intensity (%) _____ EPG (Arithmetic)**

(%) Neg Light Mod. Heavy Mean Max

Overall 55.0 44.0 35.9 12.9 6.2 104.1 4,498

Sex

Female 53.0 47.0 33.9 12.6 6.5 100.1 4,162

Male 57.6 42.4 38.5 13.3 5.8 109.3 4,498

*p*-value 0.224 0.564

Age group (years)

1 – 4 27.9 72.1 24.6 3.3 0 14.5 197

5 – 9 53.8 46.2 35.4 12.0 6.3 143.6 4,162

10 – 14 66.7 33.3 38.2 16.0 12.5 152.1 4,498

15 – 19 73.5 26.5 39.7 25.0 8.8 124.0 874

20 – 29 65.1 34.9 43.4 13.3 8.4 103.6 1,494

30 – 39 50.7 49.3 36.0 12.0 2.7 69.6 1,978

40 – 49 44.6 55.4 32.1 10.7 1.8 78.8 3,048

**≥**50 38.7 61.3 32.3 6.5 0 23.9 375

*p*-value <0.001 <0.001

Residence

Rural 61.0 39.0 36.9 14.6 9.6 156.3 4,498

Urban 48.2 51.8 34.9 10.9 2.4 44.6 720

*p*-value <0.001 <0.001

Ethnic group

Nilo-Hamite 46.9 53.1 35.0 8.8 3.1 43.9 667

Bantu 56.1 43.9 36.5 13.8 5.8 110.3 4,498

Nilotic 54.8 45.2 37.4 12.1 5.4 72.3 874

Sudanese 69.6 30.4 31.9 20.3 17.4 292.2 3,398

*p*-value 0.016 0.001

Altitude

<1000 m 61.0 39.0 36.9 14.6 9.6 156.3 4,498

**≥**1000 m 48.2 51.8 34.9 10.9 2.4 44.6 720

*p*-value 0.001 <0.001

Mod. = prevalence of moderate infection intensity; Heavy = prevalence of heavy infection intensity; Mean = mean arithmetic egg per gram (epg); Max = maximum egg per gram (epg); *p* -value = probability value comparing infection prevalence and heavy infection intensity by sex, age groups, residence, ethnic groups, and altitude.

**Table I: *Schistosoma mansoni* infection prevalence and intensity in the 2017 in–depth study.** Study conducted in 12 purposively selected villages in Ituri province (n=707). Study participants provided two stool samples. From each sample, two Kato–Katz (KK) smears were examined (total four smears per person). KK results taken in account alone.by sex, age categories, residence, ethnic groups, and altitude.

**Sex Female Male**

**Characteristics** Prev. Heavy Mean Max Prev. Heavy Mean Max

Overall 53.0 6.5 100.1 4,162 57.6 5.8 109.3 4,498

Age group (years)

1 – 4 24.1 0 12.8 197 31.3 0 16.1 160

5 – 9 50.7 5.2 148.1 4,162 56.8 7.4 139.3 3,451

10 – 14 60.3 13.7 127.4 1,123 73.2 11.3 177.5 4,498

15 – 19 67.5 12.5 144.8 874 82.1 3.6 94.3 732

20 – 29 65.7 7.5 106.2 1,494 62.5 12.5 92.5 643

30 – 39 44.9 4.1 87.9 1,978 61.5 0 35.1 288

40 – 49 50.0 0 18.3 104 37.5 4.2 159.5 3,048

**≥**50 38.7 0 31.8 375 38.7 0 16.0 110

*p*-value 0.002 0.003 <0.001 0.001

Residence

Rural 57.9 10.7 150.4 4,162 65.2 8.1 164.2 4,498

Urban 47.3 1.7 40.5 636 49.3 3.4 49.5 720

*p*-value 0.034 0.002 0.005 0.012

Ethnic group

Nilo-Hamite 46.9 5.1 53.4 667 46.8 0 29.0 312

Bantu 54.2 6.2 105.3 4,162 58.7 5.3 117.1 4,498

Nilotic 52.3 6.8 82.9 874 57.7 3.9 60.4 732

Sudanese 66.7 12.1 257.3 3,398 72.2 22.2 324.1 3,048

*p*-value 0.257 0.563 0.103 0.001

Altitude

<1000 m 57.9 10.7 150.4 4,162 65.2 8.1 164.2 4,498

**≥**1000 m 47.3 1.7 40.5 636 49.3 3.4 49.5 720

*p*-value 0.034 0.002 0.005 0.012

Prev. = infection prevalence; Heavy = prevalence of heavy infection intensity; Mean = mean arithmetic egg per gram (epg); Max = maximum egg per gram (epg); *p* -value = probability value comparing infection prevalence and heavy infection intensity among females and males by age groups, residence, ethnic groups, and altitude.

**Table J: *Schistosoma mansoni* infection prevalence in the 2017 in-depth study.** Study conducted in 12 villages in Ituri province (n=707). From each sample, two Kato–Katz (KK) smears were examined (total four smears per person). In addition, each participant provided a urine sample for point–of–care circulating cathodic antigen (POC–CCA) test. Results of the 4 different diagnostic approaches (1KK, 2KK, POC-CCA, and combined 2KK+POC-CCA).

**Diagnostic approach 1KK 2KK CCA 2KK+CCA**

**Entity/** Prevalence Total n (%) n (%) n (%) n (%)

**Villages**

Overall 707 272 (38.5) 389 (55.0) 442 (62.5) 517 (73.1)

Bankoko 31 1 (3.2) 3 (9.7) 10 (32.3) 10 (32.3)

Lumumba 36 7 (19.4) 12 (33.3) 18 (50.0) 21 (58.3)

Kindia 84 18 (21.5) 32 (38.1) 60 (71.4) 61 (72.6)

Mangenengene41 9 (22.0) 20 (48.8) 16 (39.2) 28 (68.3)

Simbilyabo 77 23 (29.9) 27 (35.1) 33 (42.9) 43 (55.8)

Sukisa 59 23 (39.0) 40 (67.8) 29 (49.2) 46 (78.0)

Kadjugi 70 28 (40.0) 39 (55.7) 39 (55.7) 49 (70.0)

Gupe 119 49 (41.2) 68 (57.1) 75 (63.0) 87 (73.1)

Ngezi 74 35 (47.3) 48 (64.9) 59 (79.7) 63 (85.1)

Mandima 40 22 (55.0) 31 (77.5) 34 (85.0) 38 (95.0)

Mambau 23 14 (60.8) 19 (82.6) 19 (82.6) 20 (87.0)

Pekele 53 43 (81.1) 50 (94.3) 50 (94.3) 51 (96.2)

*p*-palue <0.001 <0.001 <0.001 <0.001

**Health districts**

Overall 707 272 (38.5) 389 (55.0) 442 (62.5) 517 (73.1)

Nia-Nia 72 10 (13.9) 23 (31.9) 26 (36.1) 38 (52.8)

Tchomia 70 28 (40.0) 39 (55.7) 39 (55.7) 49 (70.0)

Angumu 119 49 (41.2) 68 (57.1) 75 (63.0) 87 (73.1)

Lolwa 76 57 (75.0) 69 (90.8) 69 (90.8) 71 (93.4)

Mandima 40 22 (55.0) 31 (77.5) 34 (85.0) 38 (95.0)

Bunia 330 106 (32.1) 159 (48.2) 199 (60.3) 234 (70.9)

*p*-palue <0.001 <0.001 <0.001 <0.001

1KK = prevalence resulting from the examination of one stool sample (two smears); 2KK = prevalence resulting from the examination of two stool samples (four smears); CCA = point-of-care circulating cathodic antigen test; 2KK+CCA = prevalence resulting from the combination of 2KK with CCA. *p* -value = probability value comparing infection prevalence using different diagnostic approaches by villages, and health districts.

**Table K. *Schistosoma mansoni* infection prevalence and intensity in the 2017 in–depth study.** Study conducted in 12 purposively selected villages in Ituri province (n=707). Study participants provided two stool samples. From each sample, two Kato–Katz (KK) smears were examined (total four smears per person). In addition, each participant provided a urine sample for point–of–care circulating cathodic antigen (POC–CCA) test. Combined KK+POC-CCA results.

Characteristic Prevalence Infection Intensity

____________ _______________________________________

KK+CCA n (%) Light n (%) Moderate n (%) Heavy n (%)

Overall 517 (73.1) 254 (35.9) 91 (12.9) 44 (6.2)

Sex

Female 288 (72.4) 135 (33.9) 50 (12.6) 26 (6.5)

Male 229 (74.1) 119 (38.5) 41 (13.3) 18 (5.8)

*p*-value 0.603 0.564

Age categories (years)

1 – 4 38 (62.3) 15 (24.6) 2 (3.3) 0 (0)

5 – 9 106 (67.1) 56 (35.4) 19 (12.0) 10 (6.3)

10 – 14 124 (86.1) 55 (38.2) 23 (16.0) 18 (12.5)

15 – 19 55 (80.9) 27 (39.7) 17 (25.0) 6 (8.8)

20 – 29 69 (83.1) 36 (43.4) 11 (13.3) 7 (8.4)

30 – 39 50 (66.7) 27 (36.0) 9 (12.0) 2 (2.7)

40 – 49 36 (64.3) 18 (32.1) 6 (10.7) 1 (1.8)

≥50 39 (62.9) 20 (32.3) 4 (6.5) 0 (0)

*p*-value <0.001 <0.001

Residence

Rural 283 (75.1) 139 (36.9) 55 (14.6) 36 (9.6)

Urban 234 (70.9) 115 (34.9) 36 (10.9) 8 (2.4)

*p*-value 0.214 <0.001

Ethnic groups

Nilo–Hamite 110 (68.8) 56 (35.0) 14 (8.8) 5 (3.1)

Bantu 229 (73.4) 114 (36.5) 43 (13.8) 18 (5.8)

Nilotic 119 (71.7) 62 (37.4) 20 (12.1) 9 (5.4)

Sudanese 59 (85.5) 22 (31.9) 14 (20.3) 12 (17.4)

*p*-value 0.068 0.001

Altitude

<1000 m 283 (75.1) 139 (36.9) 55 (14.6) 36 (9.6)

≥1000 m 234 (70.9) 115 (34.9) 36 (10.9) 8 (2.4)

*p*-value 0.214 <0.001

* KK+CCA, combined any *S. mansoni* positive result (by Kato–Katz and/or by POC–CCA); KK+, only Kato–Katz positive result (at least one *S. mansoni* egg in at least 1 of 4 smears). *p* -value = probability value comparing infection prevalence and heavy infection intensity by sex, age groups, residence, ethnic groups, and altitude.

**Table L. *Schistosoma mansoni* infection prevalence and intensity in the 2017 in–depth study.** Study conducted in 12 purposively selected villages in Ituri province (n=707). Study participants provided two stool samples. From each sample, two Kato–Katz (KK) smears were examined (total four smears per person). In addition, each participant provided a urine sample for point–of–care circulating cathodic antigen (POC–CCA) test. Only POC-CCA results.

Characteristic Prevalence Infection Intensity

____________ _______________________________________

POC-CCA n (%) Light n (%) Moderate n (%) Heavy n (%)

Overall 442 (62.5) 254 (35.9) 91 (12.9) 44 (6.2)

Sex

Female 249 (62.6) 135 (33.9) 50 (12.6) 26 (6.5)

Male 193 (62.5) 119 (38.5) 41 (13.3) 18 (5.8)

*p*-value 0.978 0.564

Age categories (years)

1 – 4 33 (54.1) 15 (24.6) 2 (3.3) 0 (0)

5 – 9 89 (56.3) 56 (35.4) 19 (12.0) 10 (6.3)

10 – 14 110 (76.4) 55 (38.2) 23 (16.0) 18 (12.5)

15 – 19 46 (67.7) 27 (39.7) 17 (25.0) 6 (8.8)

20 – 29 61 (73.5) 36 (43.4) 11 (13.3) 7 (8.4)

30 – 39 43 (57.3) 27 (36.0) 9 (12.0) 2 (2.7)

40 – 49 31 (55.4) 18 (32.1) 6 (10.7) 1 (1.8)

≥50 29 (46.8) 20 (32.3) 4 (6.5) 0 (0)

*p*-value <0.001 <0.001

Residence

Rural 243 (64.5) 139 (36.9) 55 (14.6) 36 (9.6)

Urban 199 (60.3) 115 (34.9) 36 (10.9) 8 (2.4) *p*-value 0.255 <0.001

Ethnic groups

Nilo–Hamite 89 (55.6) 56 (35.0) 14 (8.8) 5 (3.1)

Bantu 195 (62.5) 114 (36.5) 43 (13.8) 18 (5.8)

Nilotic 104 (62.7) 62 (37.4) 20 (12.1) 9 (5.4)

Sudanese 54 (78.3) 22 (31.9) 14 (20.3) 12 (17.4) *p*-value 0.014 0.001

Altitude

≥1000 m 199 (60.3) 115 (34.9) 36 (10.9) 8 (2.4)

<1000 m 243 (64.5) 139 (36.9) 55 (14.6) 36 (9.6)

*p*-value 0.255 <0.001

* POC–CCA (point-of-care circulating cathodic antigen) test alone; Infection intensity only for Kato–Katz positive result (at least one S. mansoni egg in at least 1 of 4 smears). *p* -value = probability value comparing infection prevalence and heavy infection intensity by sex, age groups, residence, ethnic groups, and altitude.

**Table M: *Schistosoma mansoni* infection prevalence (%) in the 2017 in–depth study.** Study conducted in 12 purposively selected villages in Ituri province (n=707). Study participants provided two stool samples. From each sample, one to two Kato–Katz (KK) smears were examined (total two to four smears per person). In addition, each participant provided a urine sample for point–of–care circulating cathodic antigen (POC–CCA) test. POC-CCA results. Prevalence of the three diagnostic approaches by sex.

**Variable Females’ prevalence (%) Males’ prevalence (%)_________**

2KK+CCA CCA 2KK 2KK+CCA CCA 2KK

**Overall** 72.4 62.6 53.2 74.1 62.5 57.6

**Age group**

**(years)**

1 – 4 62.1 58.6 24.1 62.5 50.0 31.3

5 – 9 61.0 50.7 50.7 72.8 61.7 56.8

10 – 14 84.9 75.3 60.3 87.3 77.5 73.2

15 – 19 75.0 67.5 67.5 89.3 67.9 82.1

20 – 29 80.6 68.7 65.7 93.8 93.8 62.5

30 – 39 67.4 61.2 44.9 65.4 50.0 61.5

40 – 49 68.8 56.3 50.0 58.3 54.2 37.5

**≥**50 71.0 54.8 38.7 54.8 38.7 38.7

*p* -value 0.030 0.077 0.002 0.001 0.001 <0.001

**Residence**

Rural 73.6 64.4 57.9 77.0 64.6 65.2

Urban 70.9 60.4 47.3 71.0 60.1 49.3

*p* -value 0.544 0.422 0.034 0.223 0.419 0.005

**Ethnic group**

Nilo-Hamite 67.4 56.1 46.9 71.0 54.8 46.8

Bantu 74.3 63.1 54.2 72.2 61.7 58.7

Nilotic 69.3 63.4 52.3 74.4 64.1 57.7

Sudanese 84.9 81.8 66.7 86.1 75.0 72.2

*p* -value 0.208 0.071 0.257 0.350 0.253 0.103

**Altitude**

<1000 m 73.6 64.4 57.9 77.0 64.6 65.2

**≥**1000 m 70.9 60.4 47.3 71.0 60.1 49.3

*p* -value 0.544 0.422 0.034 0.223 0.419 0.005

2KK = prevalence resulting from the examination of two stool samples (four smears); CCA = point-of-care circulating cathodic antigen test; 2KK+CCA = prevalence resulting from the combination of 2KK with CCA. *p* -value = probability value comparing infection prevalence using different diagnostic approaches among females and males by age groups, residence, ethnic groups, and altitude.

**Table N. Demographic and socioeconomic risk factors for *Schistosoma mansoni* infection (2017 in–depth study).** Results obtained with the univariate analysis of risk groups for infection with *S. mansoni* among participants from 12 villages in Ituri province in 2017 (n=707).

Risk factors Infected OR (95% CI) χ² p

n=517 (%)

Demographics

Gender*

Female 288 (72.4) 1.0

Male 229 (74.1) 1.09 (0.78–1.53) 0.27 0.603

Age categories (year)*

1 – 4 38 (62.3) 1.0

5 – 9 106 (67.1) 1.23 (0.67–2.29) 0.45 0.504

10 – 14 124 (86.1) 3.75 (1.81–7.78) 14.59 <0.001

15 – 19 55 (80.9) 2.56 (1.13–5.79) 5.48 0.019

20 – 29 69 (83.1) 2.98 (1.34–6.63) 7.94 0.005

30 – 39 50 (66.7) 1.21 (0.60–2.46) 0.28 0.597

40 – 49 36 (64.3) 1.09 (0.51–2.32) 0.05 0.824

≥50 39 (62.9) 1.03 (0.49–2.14) 0.00 0.945

Residence

Urban 234 (70.9) 1.0

Rural 283 (75.1) 1.24 (0.88–1.72) 1.55 0.214

Ethnic groups

Nilo–Hamites 110 (68.8) 1.0

Bantu 229 (73.4) 1.25 (0.82–1.91) 1.13 0.287

Nilotic 119 (71.7) 1.15 (0.72–1.85) 0.34 0.563

Sudanese 59 (85.5) 2.68 (1.25–5.75) 6.97 0.008

Body mass index

Obese 37 (58.7) 1.0

Overweight 18 (75.0) 2.11 (0.72–6.14) 1.96 0.162

Normal weight 204 (75.3) 2.14 (1.20–3.82) 6.94 0.008

Underweight 258 (73.9) 1.99 (1.14–3.49) 6.05 0.014

Year of residence*

1 – 4 166 (67.8) 1.0

5 – 9 180 (70.3) 1.13 (0.77–1.65) 0.38 0.536

≥10 171 (83.0) 2.32 (1.47–3.68) 13.76 <0.001

Socioeconomic status

Housing*

Good 23 (57.5) 1.0

Not good 494 (74.1) 2.11 (1.10–4.06) 5.26 0.022

Shoes possession*

Owning shoes 194 (68.3) 1.0

Not owning shoes 323 (76.4) 1.50 (1.07–2.10) 5.59 0.018

*Included in multi variable logistic regression analysis.

n: number; OR: odds ratio; CI: confidence interval.

**Table O. Environmental risk factors for *Schistosoma mansoni* infection (2017 in–depth study).** Results of the univariate analysis of risk groups for infection with *S. mansoni* among participants from 12 villages in Ituri province (n=707).

Risk factors Infected OR (95% CI) χ² p

n=517 (%)

Geographic risk factors

Health District*

Nia–Nia 38 (52.7) 1.0

Tchomia 49 (70.0) 2.09 (1.03–4.22) 4.41 0.036

Bunia 234 (70.9) 2.18 (1.29–3.69) 8.86 0.003

Angumu 87 (73.1) 2.43 (1.30–4.57) 8.16 0.004

Lolwa 71 (93.4) 12.71 (4.02–40.18) 31.26 <0.001

Mandima 38 (95.0) 17.00 (3.21–89.99) 20.83 <0.001

Region

Central region 234 (70.9) 1.0

Eastern region 136 (72.0) 1.05 (0.71–1.57) 0.06 0.800

Southwestern region 147 (78.2) 1.47 (0.97–2.24) 3.26 0.071

Environmental risk factors

Elevation level

High (≥1000 m) 234 (70.9) 1.0

Low (<1000 m) 283 (75.1) 1.24 (0.89–1.72) 1.55 0.214

Vegetation

Savanna 370 (71.3) 1.0

Forest 147 (78.2) 1.44 (0.97–2.15) 3.34 0.068

Distance to the nearby water bodies*

Far (≥500 m) 166 (64.8) 1.0

Near (<500 m) 351 (77.8) 1.90 (1.35–2.68) 13.99 <0.001

*Included in multi variable logistic regression analysis.

n: number; OR: odds ratio; CI: confidence interval.

**Table P. Behavioural risk factors for *Schistosoma mansoni* infection (2017 in–depth study).** Results of the univariate analysis of risk groups for infection with *Schistosoma mansoni* among participants from 12 villages in Ituri province (n=707).

Risk factors Infected OR (95% CI) χ² p

n=517 (%)

Presence of latrine in the household*

Yes 418 (70.9) 1.0

No 99 (84.6) 2.26 (1.32–3.87) 9.40 0.002

Declare using a latrine

Yes 420 (72.2) 1.0

No 97 (77.6) 1.34 (0.84–2.11) 1.54 0.214

Use of soap for washing hands

Yes 242 (71.4) 1.0

No 275 (74.7) 1.19 (0.85–1.65) 1.00 0.317

Bathing in streams*

No 212 (70.2) 1.0

Yes 305 (75.3) 1.29 (0.93–1.81) 2.30 0.130

Bathing in the lake

No 501 (72.9) 1.0

Yes 16 (80.0) 1.49 (0.49–4.50) 0.49 0.482

Swimming in water bodies*

No 452 (71.8) 1.0

Yes 65 (84.4) 2.13 (1.12–4.06) 5.60 0.018

Washing clothing in water bodies*

No 100 (65.4) 1.0

Yes 417 (75.3) 1.61 (1.10–2.38) 5.98 0.014

Washing dishes in water bodies

No 505 (72.9) 1.0

Yes 12 (85.7) 2.23 (0.49–10.10) 1.15 0.284

Fishing*

No 468 (72.1) 1.0

Yes 49 (84.5) 2.11 (1.01–4.39) 4.14 0.042

Farming*

No 211 (68.1) 1.0

Yes 306 (77.1) 1.58 (1.13–2.21) 7.19 0.007

Trading

No 490 (73.2) 1.0

Yes 27 (71.1) 0.90 (0.44–1.85) 0.09 0.767

Cleaning motorcycles in water bodies

No 498 (72.4) 1.0

Yes 19 (100.0) – – 7.17 0.007

*Included in multi variable logistic regression analysis.

n: number; OR: odds ratio; CI: confidence interval.

**Table Q. Family and individual risk factors for *Schistosoma mansoni* infection (2017 in–depth study).** Results of the univariate analysis of risk groups for infection with *Schistosoma mansoni* among participants from 12 villages in Ituri province (n=707).

Risk factors Infected OR (95% CI) χ² p

n=517 (%)

Family history of schistosomiasis*

No 433 (74.4) 1.0

Yes 84 (67.2) 0.71 (0.46–1.07) 2.71 0.100

Prior treatment for schistosomiasis*

No 431 (72.1) 1.0

Yes 86 (78.9) 1.45 (0.88–2.38) 2.18 0.140

Knowledge of schistosomiasis as a disease

No 382 (71.9) 1.0

Yes 135 (76.7) 1.28 (0.86–1.91) 1.52 0.217

Knowledge of the transmission of schistosomiasis

No 397 (72.5) 1.0

Yes 120 (75.5) 1.17 (0.78–1.76) 0.57 0.449

Knowledge of the prevention of schistosomiasis*

No 392 (71.5) 1.0

Yes 125 (78.6) 1.46 (0.96–2.23) 3.14 0.076

Knowledge of praziquantel as treatment of schistosomiasis*

No 482 (74.3) 1.0

Yes 35 (60.3) 0.53 (0.30–0.92) 5.24 0.022

*Included in multi variable logistic regression analysis.

n: number; OR: odds ratio; CI: confidence interval.

**Table R. Risk factors for *Schistosoma mansoni* infection (2017 in–depth study).** Results of the multivariable analysis of risk groups for infection with *Schistosoma mansoni* among participants from 12 villages in Ituri province (n=707).

Risk factors OR (95% CI) Std. Err. z p–value

Demographic risk factors

Age groups 0.92 (0.83–1.02) 0.049 –1.53 0.127

Gender (Male/Female) 1.20 (0.82–1.76) 0.234 0.94 0.348

Socioeconomic risk factors

Poor housing (Yes/No) 2.10 (1.02–4.35) 0.779 2.01 0.044

Lack of shoes (Yes/No) 1.28 (0.86–1.91) 0.260 1.21 0.226

Environmental risk factor

Health district 1.13 (1.04–1.23) 0.049 2.83 0.005

Water (<500m/≥500m) 1.72 (1.18–2.49) 0.325 2.85 0.004

Year of residence (≥10/<10) 1.41 (1.11–1.79) 0.174 2.78 0.005

Behavioural risk factors

Lack of latrine (Yes/No) 2.00 (1.11–3.60) 0.602 2.30 0.022

Bathing (Yes/No) 0.88 (0.60–1.30) 0.175 –0.62 0.537

Swimming (Yes/No) 2.53 (1.20–5.32) 0.959 2.45 0.014

Washing (Yes/No) 1.75 (1.10–2.78) 0.412 2.37 0.018

Fishing (Yes/No) 1.95 (0.85–4.48) 0.826 1.58 0.115

Farming (Yes/No) 1.43 (0.94–2.18) 0.306 1.66 0.096

Knowledge

Family history (Yes/No) 0.52 (0.29–0.94) 0.157 –2.17 0.030

Prior treatment (Yes/No) 1.63 (0.90–2.95) 0.493 1.61 0.108

Know prevention (Yes/No) 2.35 (1.36–4.08) 0.660 3.05 0.002

Know praziquantel (Yes/No) 0.33 (0.16–0.69) 0.125 –2.93 0.003

OR: odds ratio; CI: confidence interval.
